# A pipeline for validation of *Serendipita indica* effector-like sRNA suggests cross-kingdom communication in the symbiosis with Arabidopsis

**DOI:** 10.1093/jxb/erae515

**Published:** 2024-12-26

**Authors:** Sabrine Nasfi, Saba Shahbazi, Katharina Bitterlich, Ena Šečić, Karl-Heinz Kogel, Jens Steinbrenner

**Affiliations:** Institute of Phytopathology, Research Centre for BioSystems, Land Use and Nutrition, Justus-Liebig-University Giessen, Heinrich-Buff-Ring 26, D-35392 Giessen, Germany; Institute of Phytopathology, Research Centre for BioSystems, Land Use and Nutrition, Justus-Liebig-University Giessen, Heinrich-Buff-Ring 26, D-35392 Giessen, Germany; Institute of Phytopathology, Research Centre for BioSystems, Land Use and Nutrition, Justus-Liebig-University Giessen, Heinrich-Buff-Ring 26, D-35392 Giessen, Germany; Institute of Phytopathology, Research Centre for BioSystems, Land Use and Nutrition, Justus-Liebig-University Giessen, Heinrich-Buff-Ring 26, D-35392 Giessen, Germany; Institute of Phytopathology, Research Centre for BioSystems, Land Use and Nutrition, Justus-Liebig-University Giessen, Heinrich-Buff-Ring 26, D-35392 Giessen, Germany; Institut de Biologie Moléculaire des Plantes, CNRS, Université de Strasbourg, 12 rue du Général Zimmer, 67084 Strasbourg, France; Institute of Phytopathology, Research Centre for BioSystems, Land Use and Nutrition, Justus-Liebig-University Giessen, Heinrich-Buff-Ring 26, D-35392 Giessen, Germany; University of Warwick, UK

**Keywords:** AGO1-IP, Arabidopsis roots, cross-kingdom communication, mutualism, *Piriformospora*, 5ʹ-RLM-RACE, RNA interference, *Serendipita indica*, small RNA, symbiosis

## Abstract

Bidirectional communication between pathogenic microbes and their plant hosts via small RNA (sRNA)-mediated cross-kingdom RNAi (ckRNAi) is a key element for successful host colonization. Whether mutualistic fungi of the *Serendipitaceae* family, known for their extremely broad host range, use sRNAs to colonize plant roots is still under debate. To address this question, we developed a pipeline to validate the accumulation, translocation, and activity of fungal sRNAs in post-transcriptional silencing of *Arabidopsis thaliana* genes. Using stem–loop quantitative reverse transcription–PCR, we detected the expression of a specific set of *Serendipita indica* (*Si*) sRNAs, targeting host genes involved in cell wall organization, hormonal signalling regulation, immunity, and gene regulation. To confirm the gene silencing activity of these sRNAs in plant cells, *Si*sRNAs were transiently expressed in protoplasts. Stem–loop PCR confirmed sRNA expression and accumulation, while qPCR validated post-transcriptional gene silencing of their predicted target genes. Furthermore, Arabidopsis ARGONAUTE 1 immunoprecipitation revealed the loading of fungal *Si*sRNAs into the plant RNAi machinery, suggesting the translocation of *Si*sRNA from the fungus into root cells. In conclusion, this study provides a blueprint for rapid selection and analysis of sRNA effectors and further supports the model of cross-kingdom communication in the Sebacinoid symbiosis.

## Teaser

A blueprint method facilitates the selection and analysis of small RNAs from the beneficial fungus *Serendipita indica* , demonstrating their involvement in Arabidopsis gene silencing and suggesting their translocation during mutualistic interaction.

## Introduction

RNAi is a regulatory process in most eukaryotes in which gene expression is silenced at the transcriptional or post-transcriptional level through the action of small RNA (sRNA). This process is associated with the control of genome stability, developmental processes, and responses to biotic and abiotic stresses (Zhan and Meyers, 2023). Two main types of sRNAs predominate in plants. miRNAs are typically 21 and 22 nucleotides (nt) in length and are processed from primary miRNAs (pri-miRNAs) containing a stem–loop structure encoded by miRNA (*MIR*) genes (Reinhart *et al.*, 2002; Meyers and Axtell, 2019). In contrast siRNAs are RNA molecules of 20–24 nt in length produced from longer endogenous RNA templates or from exogenous RNA sequences (e.g. viruses or transgenes). In plants and fungi, both miRNA and siRNA precursors are processed by RNase III-like endonucleases named DICER-like (DCL) proteins. One strand of these sRNA duplexes is then bound by ARGONAUTE (AGO) proteins and incorporated into the RNA-Induced Silencing Complex (RISC), where they target complementary mRNA transcripts to induce post-transcriptional gene silencing (PTGS) (Iwakawa and Tomari, 2022). Plant sRNAs can also move from cell to cell via plasmodesmata and systemically through the phloem, acting in a cell non-autonomous manner. Additionally, they can translocate between interacting organisms (Zhan and Meyers, 2023). For example, sRNAs are transferred bidirectionally between plant hosts and pathogenic microbes to modulate defence and virulence, a phenomenon called cross-kingdom RNAi (ckRNAi) (for reviews, see Wang *et al.*, 2017; Göhre and Weiberg, 2023; Mahanty *et al.*, 2023). ckRNAi in plant–pathogen interactions was discovered in 2013 when the necrotrophic fungus *Botrytis cinerea* was shown to induce silencing of host defence genes in *Solanum lycopersicum* and *Arabidopsis thaliana* (*At*) (Weiberg *et al.*, 2013). Since then, several studies have supported the existence of ckRNAi and also showed that the exchange of sRNA effectors can be bidirectional (Zhang *et al.*, 2016; Cai *et al.*, 2018).

Already in 2006, Huang and colleagues reported reduced nematode infectivity when root-knot nematodes (RKNs) fed on roots of Arabidopsis plants expressing dsRNA targeting the *16D10* gene, which encodes a conserved secreted RKN root growth-stimulating peptide known to be involved in parasitism (Huang *et al.*, 2006). Later, it was shown that sRNAs can indeed move from a plant to an attacking pathogen in the interaction of barley and wheat with the powdery mildew fungus (*Blumeria graminis*) (Nowara *et al.*, 2010). Transient transformation of leaf epidermal cells with a plasmid overexpressing dsRNA directed against the fungal effector *AVRA10* enhanced resistance to *B. graminis* in the absence of the powdery resistance gene *Mla10*, a technique termed host-induced gene silencing (HIGS). Since then, numerous reports have shown that HIGS is an efficient strategy for pest and disease control in crops (Koch *et al.*, 2013; Cai *et al.*, 2018; Liu *et al.*, 2020; Sang and Kim, 2020; Zand Karimi and Innes, 2022).

Although ckRNAi and related artificial processes such as HIGS were discovered in plants >18 years ago, the molecular mechanisms are still not fully understood. For instance, the bidirectional transfer of RNAs in extracellular vesicles is still a topic of debate (Rutter and Innes, 2018; He *et al.*, 2021a, b; Nasfi and Kogel, 2022), and only in recent years have a growing number of studies shed light on ckRNAi in plant–symbiont interactions (Qiao *et al.*, 2023).

ckRNAi in mycorrhizal symbioses seems to include miRNAs. For example, Pmic_miR-8, an miRNA from the ectomycorrhizal fungus *Pisolithus microcarpus*, enhanced the colonization of its host *Eucalyptus grandis*, indicating its role in the beneficial ectomycorrhizal symbiosis (Wong-Bajracharya *et al.*, 2022). Moreover, sRNA Rir2216 from the arbuscular mycorrhiza fungus (AMF) *Rhizophagus irregularis* targets the WRKY transcription factor MtWRKY69 in its host *Medicago truncatula*. Consistent with ckRNAi, Rir2216 was loaded into an MtAGO1 silencing complex, leading to the cleavage of a host target transcript encoding MtWRKY69 (Silvestri *et al.*, 2024). Moreover, tRNA-derived sRNA fragments (tRFs) identified in root nodule symbioses between soybean and the bacterial symbiont *Bradyrhizobium japonicum* have been shown to hijack *Glycine max* AGO1. These tRFs act as positive regulators of rhizobial infection and nodule formation (Ren *et al.*, 2019), further emphasizing the existence of ckRNAi in mutualistic plant–microbe interactions.

Understanding ckRNAi depends on the identification and functional validation of translocated sRNA (sRNA effectors), which requires tools to study the mode of action of numerous sRNAs derived from extensive sRNA sequencing data. In this context, we present a pipeline for validation of sRNA effectors by studying the interaction between the mutualistic basidiomycete fungus *Serendipita indica* (*Si*) and the dicot model plant *At*. The fungus was originally isolated from the roots of the shrubs *Prosopis juliflora* and *Ziziphus nummularia* in the Indian Thar desert (Verma *et al.*, 1998). Its beneficial activity includes plant growth promotion, enhanced plant resistance, enhanced nitrate and phosphate delivery, promotion of adventitious root and root hair formation, early flowering, support of higher seed yield, alteration in secondary metabolites, and hardening of tissue-cultured plants (Zuccaro *et al.*, 2011; Qiang *et al.*, 2012; Glaeser *et al.*, 2016; Weiß *et al.*, 2016; Xu *et al.*, 2018; Verma *et al.*, 2022; Galli *et al.*, 2024). Moreover, the fungus transfers protein effectors to host cells to exploit the host’s metabolism and promote microbial colonization (Akum *et al.*, 2015; Osborne *et al.*, 2023). To further explore the molecular basis of the mutualistic interaction formed by *Si* with a wide range of plants (Sebacinalean symbiosis), we recently demonstrated the global change in sRNA profiles in the interaction of the beneficial fungus and the grass model *Brachypodium distachyon* (*Bd*) (Šečić *et al.*, 2021). Among *Bd*- and *Si*-generated sRNAs with putative functions in the interacting organism, we found proteins involved in cell wall organization, hormonal signalling regulation, and immunity as potential targets of putative fungal effector sRNAs.

Building upon the findings from our prior work, we have selected a set of fungal sRNAs with interesting predicted *At* targets to further investigate their activity in the *Si–At* interaction. We developed a functional protoplast assay to validate these potential fungal effector sRNAs and assessed their gene silencing activity. Using stem–loop quantitative reverse transcription–PCR (RT–qPCR), we confirmed sRNA transformation and accumulation in plant protoplasts and recorded the down-regulation of predicted host target genes via qPCR. Additionally, we investigated the capability of *Si*sRNA candidates in mediating the degradation of host mRNA by 5ʹ-RNA ligation-mediated (RLM)-RACE. Finally, *At*AGO1 immunoprecipitation assay confirmed the loading of fungal *Si*sRNAs into the plant’s RNAi machinery. The data support the existence of naturally occurring ckRNAi between the beneficial fungus *Serendipita indica* and *Arabidopsis thaliana.*

## Materials and methods

### *Si*sRNA selection and Arabidopsis target prediction

The *Si*sRNAs selected from Šečić *et al.* (2021) and their predicted *At* target genes are summarized and detailed in Supplementary Table S1 and Supplementary Dataset S1.

### Plants, fungi, and plant inoculation

For interaction studies of *At* roots and *Si* (IPAZ-11827, Institute of Phytopathology, Giessen, Germany), *At* plants of ecotype Columbia-0 (Col-0) were grown on vertical square Petri dishes on an ATS medium (Lincoln *et al.*, 1990) without sucrose and supplemented with 4.5 g l^–1^ Gelrite (Duchefa #G1101) in a 22 °C day/18 °C night cycle (8 h of light). Roots of 14-day-old plants were inoculated with 1 ml of a suspension of 500 000 *Si* chlamydospores ml^–1^ in aqueous 0.002% Tween-20 per Petri dish as described in Jacobs *et al.* (2011). For the protoplast experiment, *At* plants were cultivated in a 4:1 ratio of T-type soil (F.-E. Typ Nullerde, Hawita) and crystal quartz sand mixture for 4–5 weeks in a growth chamber in a 19 °C day/18 °C night (9 h of light, 150 μmol photons m^–2^ s^–1^), and a constant relative humidity of 60%. *Si* was grown in axenic culture on complete medium (Pontecorvo *et al.*, 1953) for 4 weeks in daylight under sterile conditions with shaking at 100 rpm.

### Plasmid construct

#### Digestion–ligation of the *At*MIR390a distal stem–loop

The Arabidopsis MIR390a distal stem–loop, containing a *ccd*B cassette flanked by two *Bsa*I sites (*At*MIR390a-B/c), was excised from the pMDC32B-*At*MIR390a-B/c vector (Plasmid #51776, https://www.addgene.org, Carbonell *et al.*, 2014) using *Eco*RI and *Hin*dIII Fast digest restriction enzymes (Thermo Fisher Scientific, FD0274 & FD0505). The pUC18 vector backbone (plasmid #50004) was similarly digested with the same enzymes. The *At*MIR390a-B/c fragment was then ligated into the pUC18 vector backbone using T4 DNA Ligase (Thermo Fischer Scientific, EL0011) generating the pUC18-*At*MIR390a-B/c protoplast expression vector for MIR390a-based 21 or 22 nt sRNAs. Digestion was carried out at 37 °C for 1 h, followed by enzyme inactivation at 80 °C for 10 min. The ligation was performed overnight at 16 °C.

#### Digestion ligation of the red fluorescent protein cassette

The red fluorescent protein (RFP) cassette was excised from the pBeaconRFP_GR vector (https://gatewayvectors.vib.be/index.php/ ID:3_20, Bargmann and Birnbaum, 2009) using *Nde*I restriction enzyme (NEB, R0111S). The pUC18-*At*MIR390a-B/c vector, which contains an *Nde*I restriction site, was similarly digested. The RFP cassette was then ligated into the *Nde*I site of the pUC18-*At*MIR390a-B/c vector using T4 DNA ligase.

#### Site-directed mutagenesis

Before cloning 75-mer oligonucleotides, a third *Bsa*I site in the pUC18 backbone, which would interfere with the Golden Gate cloning of artificial miRNAs (amiRNAs) and *Si*sRNA, was removed by site-directed mutagenesis. This was achieved using Phusion High-Fidelity DNA polymerase (Thermo Fisher Scientific, F553S), the fast digest *Dpn*I endonuclease (Thermo Fisher Scientific, FD1703), and custom-designed site-directed mutagenesis primers (pUC18-Mut-Fwd and pUC18-Mut-Rev) following the manufacturer’s protocol.

#### Direct cloning of amiRNAs and *Si*sRNAs

The 75-mer oligonucleotides comprising the 21 nt sequences of amiRNAs and *Si*sRNAs were designed using the P-SAMS web-tool (http://p-sams.carringtonlab.org/) as described by Fahlgren *et al.* (2016), Carbonell *et al.* (2014), and Carbonell (2017a, 2019). Forward and reverse 75-mer oligonucleotides were diluted to 100 µM and annealed using a thermocycler under the following conditions: 5 min at 94 °C, followed by cooling at 0.05 °C s^–1^ to 20 °C. Prior to cloning, the annealed 75-mer oligonucleotides were further diluted to 0.15 µM. These oligonucleotides were then cloned into the *Bsa*I sites of the pUC18-*At*MIR390a-B/c vector using the Golden Gate strategy. A single digestion–ligation reaction was performed with *BsaI-HF*^*®*^*v2* (NEB, R3733) and T4 DNA ligase (Thermo Fischer Scientific, EL0011), replacing the ccdB cassette, allowing for the selection of 75-mer-positive clones.

An *in silico* cloning design was performed using Snapgene software (https://www.snapgene.com). The cloning strategy workflow is outlined in Supplementary Fig. S1A, while Supplementary Fig. S1B presents the final pUC18-*At*MIR390a-RFP-amiRNA/*Si*sRNA vector map. The content of the 75-mer oligonucleotides is illustrated in Supplementary Fig. S2. The designed amiRNAs, including their 5ʹ-nucleotide sequences, predicted targets, target aligned fragments, expectation values, and mode of regulation, are detailed in Supplementary Table S2. Similarly, the same information for the selected putative *Si*sRNAs cloned into the expression vector for PTGS analysis are listed in Supplementary Table S3. An alignment of amiRNAs and *Si*sRNA with their predicted *At* targets, as generated by the psRNATarget web-tool, is shown in Supplementary Fig. S3A, while Supplementary Fig. S3B provides a color-coded heatmap visualizing the sRNA–mRNA pair alignment created using RStudio. The forward and reverse 75-mer oligonucleotides for amiRNAs and *Si*sRNAs are listed in Supplementary Table S4.

### Protoplast transformation

Protoplasts were isolated using the ‘Tape–Arabidopsis Sandwich’ method (Wu *et al.*, 2009). Transformation was performed using the polyethylene glycol (PEG) method, following the transient expression of recombinant genes using the Arabidopsis mesophyll protoplast (TEAMP) approach (Yoo *et al.*, 2007), with minor modifications (WI incubation solution was replaced by the W5 solution). Since transformation efficiency was crucial for all subsequent experiments, we optimized the transformation by varying (i) protoplast concentration (10×10^4^ and 5×10^4^ protoplasts ml^–1^), (ii) plasmid pUC18-*At*MIR390a-RFP-sRNA concentration (20, 30, and 40 µg), and (ii) incubation time (24 h and 48 h). High transformation efficiency was achieved with 5×10^4^ protoplasts ml^–1^, 30 µg of plasmid, and 24 h incubation time, comparable with the efficiencies reported for *At* leaf protoplasts (Yoo *et al.*, 2007). Total cell numbers were counted using a Fuchs–Rosenthal counting chamber under an optical microscope. Protoplast transformation was checked using an epifluorescence microscope (TCS SP2 Leica), and images were acquired with Leica Application Suite (LAS) software. All counts were performed in triplicate. Transformed protoplasts were harvested by centrifugation at 100 *g* for 2 min, the supernatant was removed, and protoplasts were snap-frozen in liquid nitrogen and stored at –80 °C for further studies.

### RNA extraction and quality analysis

Total RNA was extracted from protoplasts using the QuickRNA™ Miniprep kit (Zymo Research, R1050) with an on-column DNase I treatment. Total RNAs from *Si–At* interaction grown on ATS plates and from *Si* mycellium grown in axenic culture were extracted using Direct-zol RNA Miniprep (Zymo Research, R2051) with an on-column DNase I treatment. RNA concentration was measured using a NanoDrop ND-1000 Spectrophotometer (Thermo Fisher Scientific, USA), and RNA purity was determined by assessing *A*_260_/_280_ and *A*_260_/_230_ ratios. The quality of the RNA extracted from transformed protoplasts was further checked using the Agilent 2100 Bioanalyzer Nano Chip (Agilent, Germany).

### Stem–loop end-point PCR

Designed amiRNA and *Si*sRNA sequences were used as templates to design specific stem–loop primers matching the corresponding sRNA over 6 nt at the 3ʹ end. Hairpin primers and forward primers were designed using the tool published in Adhikari *et al.* (2013) based on Varkonyi-Gasic and Hellens (2011). Stem–loop PCR was performed as described by Werner *et al.* (2021), either in duplexed stem–loop (cDNA generated from two sRNA hairpin primers simultaneously in one reaction) or multiplexed stem–loop (cDNA generated from multiple hairpin sRNA primers simultaneously in one reaction). End-point PCR was used to assess the expression of amiRNAs and *Si*sRNAs. The PCR program was optimized as follows: initial denaturation at 95 °C for 5 min; followed by 40 cycles of 95 °C for 30 s, 60 °C for 30 s, 72 °C for 30 s; with a final extension at 72 °C for 5 min and a hold at 4 °C. PCR products were separated and visualized in 2% TBE–agarose gels. Stem–loop PCR primers are listed in Supplementary Table S5.

### Stem–loop RT–qPCR

For the *Si*sRNA expression level analysis, cDNA was reverse transcribed from RNA extracted from *At* roots, either inoculated with *Si* or mock treated (non-inoculated), using hairpin primers in multiplexed stem–loop to target respective *Si*sRNAs, as well as two endogenous housekeeping miRNAs, *At*miR159a and *At*miR166a. To assess *Si*sRNA and amiRNA accumulation in protoplasts, cDNA was reverse transcribed using RNA extracted from transformed and control (non-transformed) protoplasts, using hairpin primers in duplexed stem–loop to target the respective amiRNAs or *Si*sRNAs, with *At*miR159a serving as the endogenous miRNA control. Amplification efficiencies were evaluated by generating stem–loop cDNA using RNA extracted from *Si* axenic culture or from *At* mock-treated roots (non-inoculated). A four-step, 10-fold dilution series of cDNA (ranging from 15 ng µl^–1^ to 15 pg µl^–1^) was used. All *Si*sRNA amplifications showed efficiencies between 80% and 110% with an *R*^2^ value between 1 and 0.991, except for *Si*sRNA154, with an efficiency of 122%. stem–loop RT–qPCR was performed with 5 ng of cDNA on the QuantStudio 5 Real-Time PCR system (Applied Biosystems). A 2 µl aliquot of ROX (CRX reference dye, Promega, C5411) was added to 1 ml of SybrGreen as a passive reference dye that allows fluorescent normalization for qPCR data. The PCR conditions were 95 °C for 5 min, followed by 40 cycles of 95 °C for 15 s, 60 °C for 30 s, and 72 °C for 30 s, and then by a melting curve analysis. Each sample had three technical replicates. Specific forward and universal reverse stem–loop primers were used for the amplification of amiRNAs or *Si*sRNAs, and relative abundance was calculated using the ΔΔCt method (Livak and Schmittgen, 2001), normalized against the geometric mean (Vandesompele *et al.*, 2002) of *At*miR159a and/or *At*miR166a.

### PTGS detection by qPCR

The standard curve method was used to test the efficiency of the qPCR transcript primers using a serial dilution of an *At* cDNA library along with three different primer concentrations (0.4, 0.2, and 0.1 µM for each primer) and 5 µl of SybrGreen (Sigma-Aldrich). The total volume of 10 µl and three technical replicates are considered for each reaction. Prior to master mix preparation, 2 µl of ROX (CRX reference dye, Promega, C5411) were added to 1 ml of SybrGreen as a passive reference dye that allows fluorescent normalization for qPCR data. For cDNA synthesis, 500 ng of RNA samples from both transformed and control *At* protoplasts were used. qPCR was performed using the QuantStudio 5 real-time PCR system (Applied Biosystems) as described before. Fold changes in expression were calculated using the ΔΔCt method (Livak and Schmittgen, 2001), normalized against the endogenous housekeeping gene *Ubiquitin* (*UBC21, AT5G25760*). Standard errors were calculated for all mean values. All qPCR primers are listed in Supplementary Table S6.

### 5ʹ-RLM-RACE

5ʹ-RLM-RACE was performed using the FirstChoice^®^ RLM-RACE kit (Thermo Fisher Scientific) following the manufacturer’s protocol and omitting the dephosphorylation and decapping steps. A 1 µg aliquot of RNA isolated from *At* transformed and control protoplasts was used as a template and ligated to the 5ʹ-RACE adapter using T4 RNA ligase (10 U µl^–1^) (Thermo Fisher Scientific). The ligation reaction was used entirely to generate the first cDNA strand. Two rounds of nested hot-start touch-down PCR were performed using outer (first) and inner (second) 5ʹ-RLM-RACE universal primers in combination consecutively with gene-specific outer and gene-specific inner primers. PCR products were evaluated in a 1.5% agarose gel, and bands of the expected size were excised. These products were cleaned with the Wizard^®^ SV Gel and PCR Clean-Up System (Promega) and cloned with the pGEM^®^-T Vector Systems (Promega). For each band, six clones were selected for sequencing by LGC Genomics (Berlin, Germany). The oligonucleotides used are listed in Supplementary Table S7.

### Arabidopsis root transcript analysis

Arabidopsis Col-0 plants were grown on ATS plates and inoculated with *Si* chlamydospores as described above. *At* mock-treated roots (non-inoculated) were treated with water containing 0.002% Tween-20. Inoculated roots were harvested at 3 and 7 days post-inoculation (dpi), ground using a tissue lyser, and RNA was extracted using Trizol and the Zymo Quick-RNA™ Miniprep kit (Zymo research R2070), with a subsequent in-column DNase digestion. cDNA was sythesized from 500 ng of RNA using Revert Aid Reverse transcriptase. Gene transcription levels were quantified by qPCR using SYBR Green JumpStart Taq ReadyMix (Sigma Aldrich, 1003444642) with a QuantStudio5 Real-Time PCR System (Applied Biosystems). A 2 µl aliquot of ROX (CRX reference dye, Promega, C5411) was added to 1 ml of SybrGreen as a passive reference dye that allows fluorescent normalization for qPCR data. PCR conditions were as previously described. Gene expression levels were normalized to the geometric mean of two endogenous housekeeping genes (Vandesompele *et al.*, 2002), *Ubiquitin* (*UBC21*, *AT5G25760*) and *Elongation Factor-1 alpha* (*EF1α*, *AT5G60390*). Fold changes in expression were calculated using the ΔΔCt method (Livak and Schmittgen, 2001). Roots from two ATS plates were considered as one biological replicate. The results of two biological replicates are included in the data analysis. All qPCR primers are listed in Supplementary Table S6.

### T-vector cloning and sequencing

Stem–loop PCR and 5ʹ-RLM-RACE PCR amplification products were purified using the Wizard^®^ SV Gel and PCR Clean-Up System (Promega). Cloning of the different PCR products was performed as described in the pGEM^®^-T Vector system according to the manufacturer’s instructions (Promega). Sequencing was performed at LGC Genomics and analysed using the Snapgene tool (GSL Biotech, available at snapgene.com). All PCR primers are presented in Supplementary Table S8.

### AGO immunoprecipitation

*At*AGO1 co-immunoprecipitation (Co-IP) was performed following Dunker *et al.* (2021) with modifications. A 5 g aliquot of *Si* mycelium or *At* roots, inoculated or not with *Si*, was ground to a fine powder using a pre-cooled mortar and pestle. To each sample, 20 ml of immunoprecipitation extraction buffer was added, then centrifuged at 3200 *g* for 15 min at 4 °C to remove root debris. The supernatants were filtered through double-layered Miracloth and 200 µl of crude extract (CE, supernatant before antibodies) was collected for western blot analysis. To the remaining supernatant, 5 µl of anti-AGO1 polyclonal antibody (Agrisera, catalogue no. AS09527), and 200 µl of protein A agarose beads (Roche, Ref: 11719408001) were added, and the mixture was incubated for 2 h at 4 °C on a rotation wheel. After centrifugation at 200 *g* for 30 s, 200 µl of the supernatant (SN, after antibodies) was collected for further western blot analysis. The remaining supernatants were discarded, and the pelleted beads were washed three times with ice-cold IP wash buffer. The washed beads were resuspended in 1 ml of wash buffer, with 30% used for western blot analysis (IP fraction) and 70% for sRNA recovery.

### sRNA recovery from the IP fraction

IP fractions were pelleted and resuspended in 300 µl of IP wash buffer with 150 µl of RNA release buffer. Samples were incubated for 15 min at 300 rpm at 65 °C. A 450 µl aliquot of water-saturated phenol was added, and samples were vortexed for 2 min then centrifuged at 10 000 *g* at room temperature for 8 min. The upper aqueous phase containing the sRNAs was transferred into a low binding RNA tube. Then 450 µl of chloroform/isoamyl alcohol (24:1) (Carl Roth, Germany) was added to the RNA samples. The step was repeated twice. For RNA precipitation, 0.1× volume of 3 M sodium acetate, 2.5× volume of 96% ethanol, and 20 µg of RNA grade glycogen (Thermo Fisher Scientific, R0551) were added to the RNA samples and left overnight at –20 °C. Samples were pelleted at 20 000 *g* for 30 min at 4 °C and washed with 500 µl of 80% ethanol. RNA was pelleted at 20 000 *g* for 20 min at 4 °C, ethanol was removed, and pellets were air-dried until ethanol was completely evaporated. The RNA pellets were resuspended in 8 µl of diethylpyrocarbonate (DEPC)-treated water and stored at –80 °C.

### Western blot analysis

Western blot analysis was performed using a 5.5% stacking gel and a 12% resolving gel. For each sample, 20 µl of total protein, crude extract before antibodies (CE), supernatant after pelleting agarose beads (SN), and *At*AGO1 Co-IP fraction (IP) were loaded after boiling at 95 °C for 5 min. Proteins were transferred to a polyvinylidene fluoride (PVDF) blotting membrane (Carl Roth, Germany). The membrane was blocked with 5% (w/v) milk powder (Carl Roth, Germany) in phosphate-buffered saline with Tween-20 (PBS-T) for 1 h at room temperature, then probed overnight at 4 °C on a shaker with anti-AGO1 primary antibody (1:4000 dilution) (Agrisera, AS09 527). After washing, the membrane was incubated for 2 h at room temperature with mouse anti-rabbit horseradish peroxidase (HRP)-conjugated (Santa Cruz-2357) secondary antibody. For the membrane development, chemiluminescent substrates were applied according to the manufacturer’s recommendation, and images were captured using the Bio-rad ChemiDoc MP imaging system.

### Statistical analysis

For qPCR gene expression data in *Si–At* interaction and in transformed protoplasts, measurements were compared with *At* non-inoculated roots and non-transformed protoplasts, respectively. Expression values were analysed using the ΔΔCt method (Livak and Schmittgen, 2001), normalized against *UBC21* and/or *EF1α* as housekeeping genes, and visualized as a fold change of the expression level. For stem–loop qPCR for the *Si*sRNA expression level in *Si–At* interaction, and for *Si*sRNA and amiRNA accumulation in transformed protoplasts, *At*miR159a and/or *At*miR166a were used as endogenous housekeeping miRNAs and expressed as log_2_ fold change of the expression level. Significance was assessed either by a two-sided unpaired Student’s *t*-test or by a two-sided Welch’s *t*-test (α=0.05, *P<0.05, **P<0.01, ***P<0.001).

## Results

**Fig. 1.**
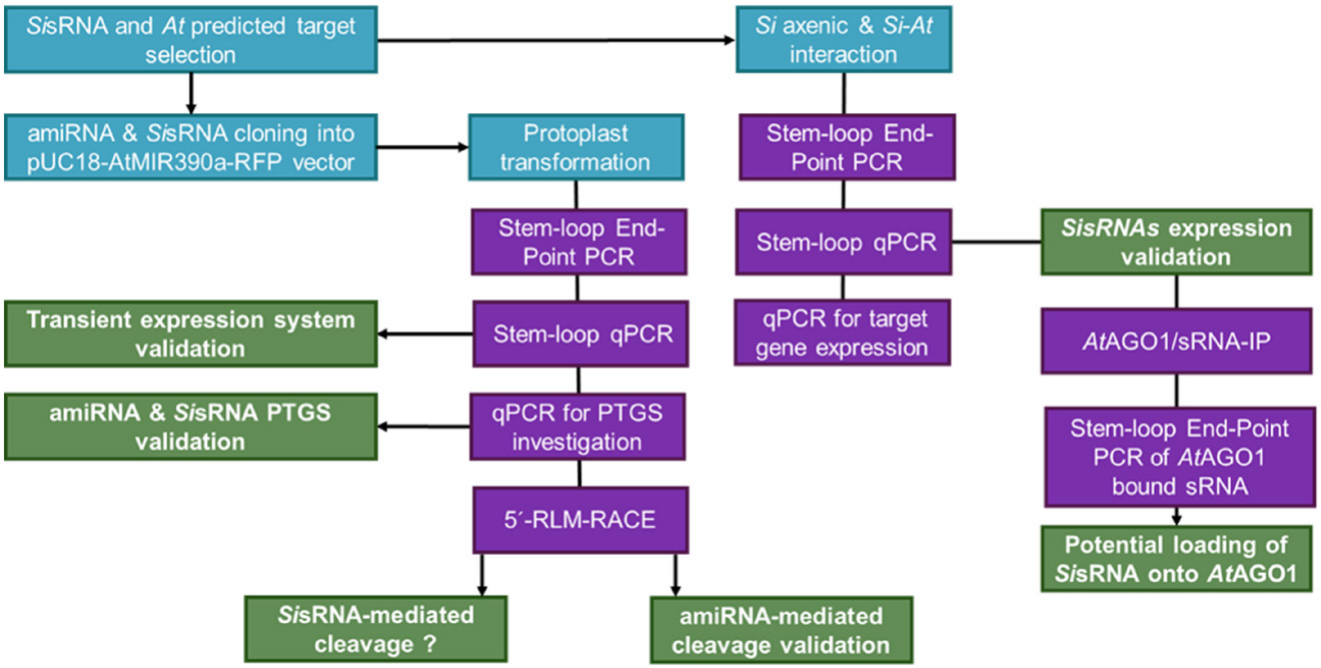
Schematic flowchart showing the methods and outputs in this study. The blue colour indicates the different input material, and purple indicates the wet-lab steps. Green represents the validated outputs of the study. For details see also the Materials and methods. amiRNAs, artificial miRNA; *At*, *Arabidopsis thaliana*; PTGS, post-transcriptional gene silencing; *Si*, *Serendipita indica*; sRNA-IP, small RNA immunoprecipitation.

We developed a pipeline for the validation of sRNA-mediated gene silencing activities in the mutualistic interaction of *Si* and *At*, comprising (i) prediction of Arabidopsis target genes for fungal sRNAs (*Si*sRNAs, using psRNATarget), (ii) detection of *Si*sRNA expression in axenic culture and *Si*-colonized *At* roots (*Si–At* interaction), (iii) validation of gene silencing activity of *Si*sRNAs in *At* protoplasts, and (iv) detection of the association of *Si*sRNAs with the *At*AGO1 protein (Fig. 1).

### Effector-like *Si*sRNA candidates are present in axenic cultures of *Serendipita indica*

**Fig. 2.**
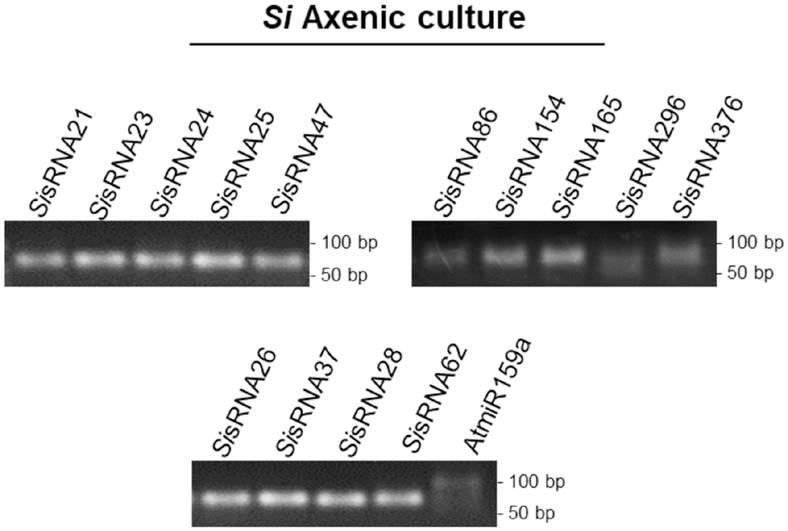
Gel electrophoresis of stem–loop PCR products of potential *Si*sRNA effector candidates in *Serendipita indica.* Multiplexed stem–loop PCR confirmed the presence of *Si*sRNAs in a 4-week-old *Si* axenic culture. A PCR product of the expected size of 62 bp (sum of the 21 bp of the sRNA sequence, loop region, plus the forward primer and the reverse primer length) is visualized in a 2% agarose gel. The plant *At*miR159a of 21 nt served as a negative control. A shorter and weaker band of *Si*sRNA296 was detected possibly due to a lower abundance of the sRNA in the sample. A weak and unspecific amplification was detected for the *At*miR159a due to the multiplexing of hairpin primers or a possible partial complementarity of the hairpin primer to a non-target sequence.

From the dataset of 412 putative *Si*sRNAs identified by sRNA sequencing in our previous study in the *Si–Bd* symbiosis (Šečić *et al.*, 2021), we selected a set of 14 potential *Si*sRNA effector candidates of 21 nt on the basis that they (i) showed a higher expression level in colonized *Bd* roots, (ii) covered all four 5ʹ-terminal nucleotides, and (iii) had interesting Arabidopsis predicted targets involved in cell wall organization, regulation of hormone signalling, immunity, and gene regulation. Using multiplexed (cDNA generated from multiple hairpin sRNA primers simultaneously in one reaction) stem–loop PCR followed by gel electrophoresis, we detected all 14 *Si*sRNAs in 4-week-old axenic *Si* cultures (Fig. 2). Next, we cloned a random subset of six out of 14 *Si*sRNAs (*Si*sRNA21, SisRNA23, *Si*sRNA24, *Si*sRNA28, *Si*sRNA154, and *Si*sRNA296) and subjected them to Sanger sequencing, which further confirmed the expected *Si*sRNA sequences.

### Effector-like *Si*sRNA candidates are detected in colonized *A. thaliana* roots

**Fig. 3.**
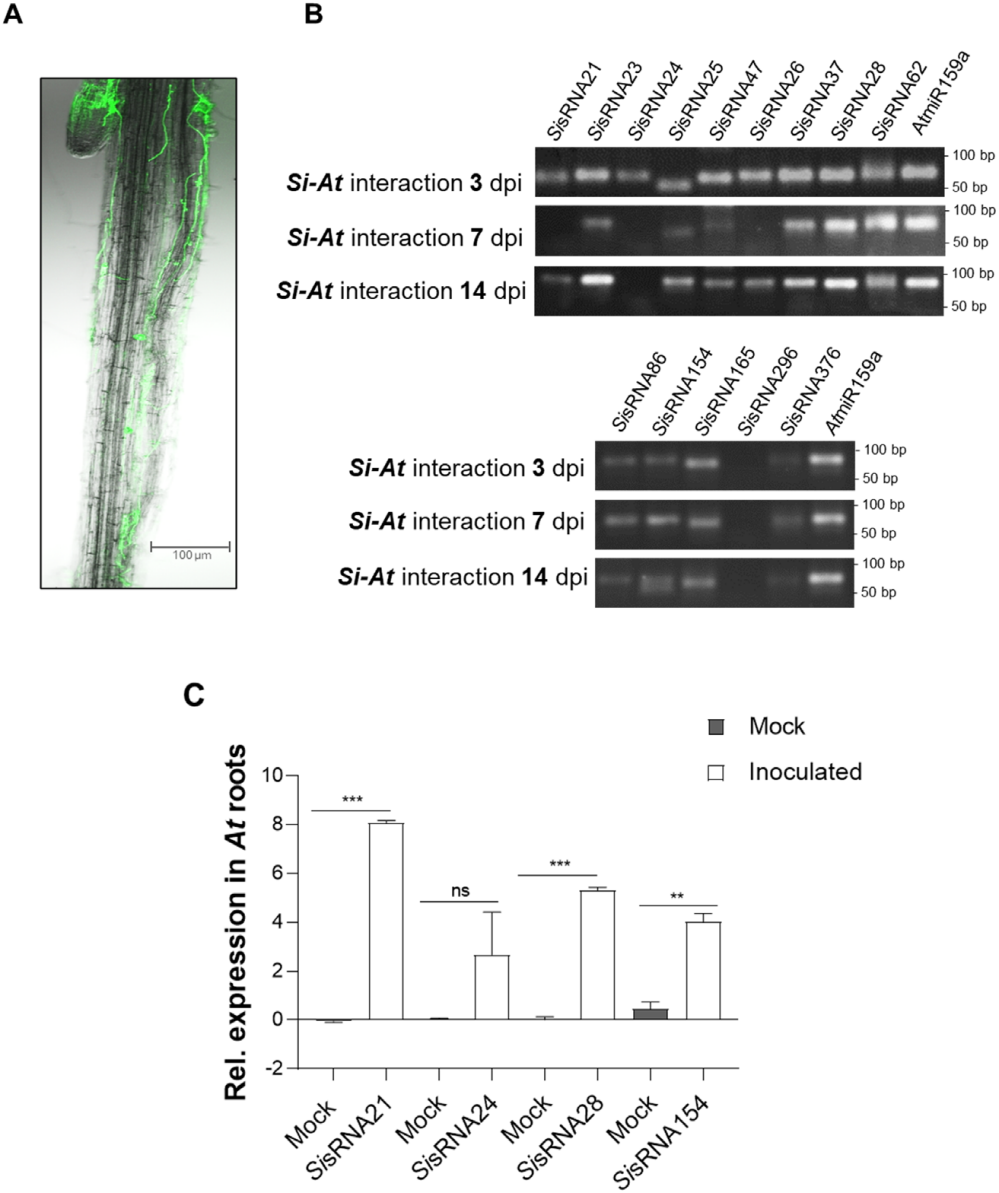
Detection of putative effector-like *Si*sRNAs in Arabidopsis roots colonized by *Serendipita indica*. (A) Root colonization pattern at 7 days post-inoculation (dpi). Fluorescence microscopy (λexc494 nm, λem515) shows green chitin-specific WGA-AF488 staining of hyphal walls in the root maturation zone. (B) Detection of *Si*sRNAs in roots at 3, 7, and 14 dpi using multiplexed stem–loop PCR. Agarose gel analysis of *Si*sRNAs of the expected size of 62 bp. Plant *At*miR159a was used as a positive control for a plant-expressed sRNA. A shorter band of the *Si*sRNA25 was detected at 3 dpi, probably due to a lower abundance of the sRNA in the samples at that time point. (C) Relative expression of four *Si*sRNAs in Arabidopsis roots inoculated with *Si* at 7 dpi. The relative amounts of *Si*sRNA21, *Si*sRNA24, *Si*sRNA28, and *Si*sRNA154 during interaction with *At* were normalized to *At*miR159a and *At*miR166a. Values represent the mean ±SE of two independent biological replicates. Asterisks indicate significant differences at *P≤0.05, **P≤0.01, and ***P≤0.001 based on Student’s *t*-test. ns=not significant.

Next, we analysed the presence of the 14 *Si*sRNAs identified in axenic culture in *Si*-colonized *At* roots. Roots of 14-day-old seedlings were inoculated with chlamydospores and harvested at 3, 7, and 14 dpi. Root colonization was confirmed after 7 dpi by confocal laser scanning microscopy using the chitin-specific dye wheat germ agglutinin linked to Alexa Fluor 488 (WGA–AF-488) (Fig. 3A) and by PCR using internal transcribed spacer (*Si*-*ITS*) primers and *Si*-specific *Ubiquitin* (*Si-Ubi*) primers (Supplementary Fig. S4). Multiplexed stem–loop PCR of total RNA extracted from *Si*-colonized *At* roots at different time points detected 13 of 14 *Si*sRNAs (Fig. 3B). Bands for *Si*sRNA21 and *Si*sRNA24 did not consistently show up at all three time points, probably because of the limitations associated with multiplexing the hairpin cDNA primers (Kramer, 2011; Varkonyi-Gasic and Hellens, 2011; Colombo *et al.*, 2013). Consistent with this notion, the expression of *Si*sRNA21 and *Si*sRNA24 was detected at 7 dpi using stem–loop PCR with less multiplexing (using four hairpin cDNA primers instead of 10 simultaneously in one reaction) (Supplementary Fig. S5). A random subset of the detected *Si*sRNAs (*Si*sRNA21, *Si*sRNA24, *Si*sRNA28, and *Si*sRNA154) were cloned and submitted for Sanger sequencing, confirming the expected *Si*sRNA sequences in *Si*-colonized *At* roots.

To assess the abundance and expression of *Si*sRNAs during the interaction with *At* roots, we selected *Si*sRNA21, *Si*sRNA24, *Si*sRNA28, and *Si*sRNA154, which were confirmed to be expressed in axenic culture and during *Si–At* interaction. Using stem–loop RT–qPCR, we detected all four *Si*sRNAs in inoculated *At* roots, while they could not be found in non-inoculated roots, indicating high abundance (Fig. 3C). These findings are consistent with the previous identification of these *Si*sRNAs in the *Si–Bd* interaction (Šečić *et al.*, 2021) (Supplementary Table S1).

### amiRNAs mediate PTGS in transformed Arabidopsis protoplasts

To validate sRNA–mRNA interactions, we developed a protoplast-based sRNA expression system. Arabidopsis protoplasts are a reproducible and cost-effective model system to validate the expression and the potential silencing activity of *Si*sRNA on predicted *At* target genes. To confirm the reliability of the system, we first used amiRNAs. amiRNAs are designed following the P.SAMS software (Fahlgren *et al.*, 2016). The software identifies 21 nt sequences from the input transcript based on high sequence complementarity to the target mRNA with a target prediction score of 0 (also called expectation value, with an expectation value of 0 being the highest level of sequence complementarity and 5 being the lowest), filters out off-targets using TargetFinder, and then designs the guide amiRNA following certain rules such as having a 5ʹ-U nucleotide, a C at position 19, and a mismatch or a bulge at position 21. The use of amiRNAs allowed the amiRNA–mRNA binding to be manipulated, as they are artificially designed to have almost complete complementarity with the mRNA target gene, unlike naturally occurring *Si*sRNAs, which have varying degrees of mismatch with their predicted target genes. To this end, we designed four amiRNAs of 21 nt, namely amir21, amir24, amir154, and amir296, that almost fully match the predicted target genes *AT5G25350*, *AT2G39020*, *AT4G32160*, and *AT2G45240*, respectively, and exhibit a U at their 5ʹ end. Target genes were chosen based on their functions with *AT5G25350* (*EIN3-BINDING F BOX PROTEIN 2*), a negative regulator of the ethylene-activated signalling pathway, *AT2G39020* (*GCN5-RELATED N-ACETYLTRANSFERASE 8*) potentially involved in development and stress response pathways, *AT4G32160* [*Phox* (*PX*) *DOMAIN-CONTAINING PROTEIN EREL1*] predicted to be involved in membrane trafficking, and *AT2G45240* (*METHIONINE AMINOPEPTIDASE 1A*) involved in post-translational modification of proteins.

**Fig. 4.**
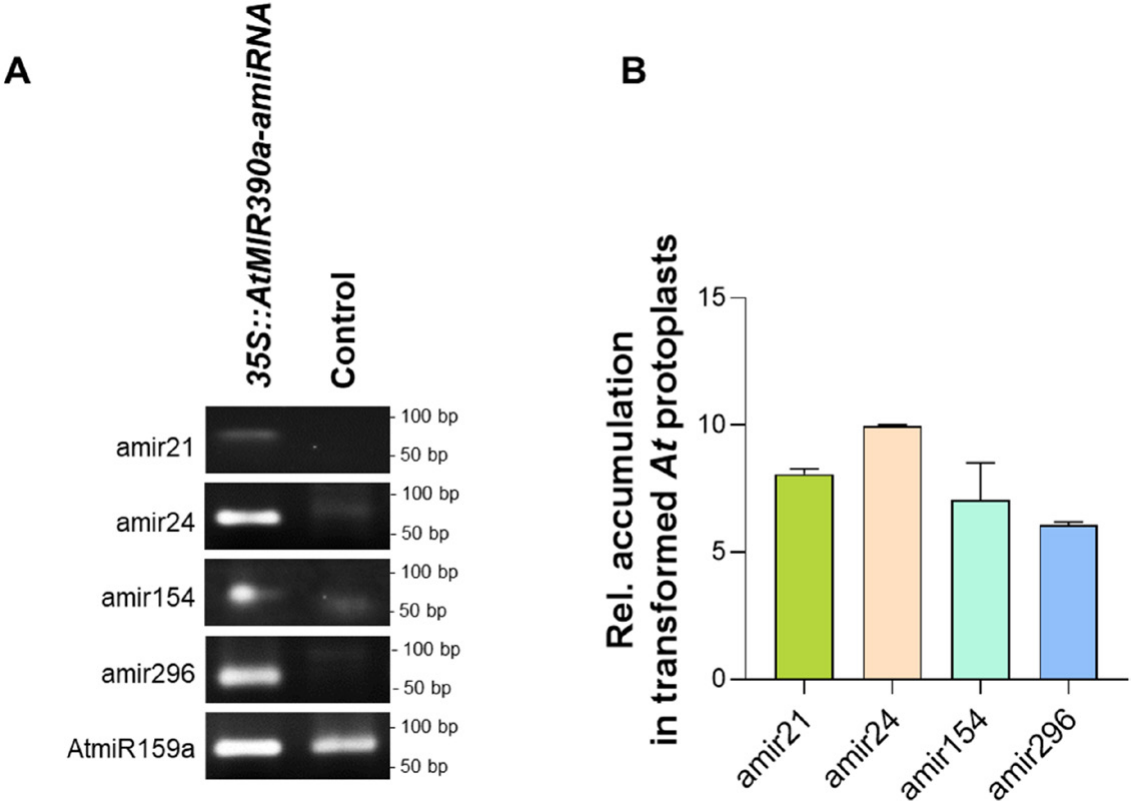
Expression and accumulation of amiRNAs in transformed Arabidopsis protoplasts. (A) Agarose gel analysis confirming expression of all four amiRNAs (amir21, amir24, amir154, and amir296) in transformed protoplasts 24 hours post-transformation (hptr), but not in control protoplasts, using duplexed stem–loop PCR. No amplification or only weak bands of primer dimers were seen in the control protoplast (without construct) samples, probably due to the high number of cycles in the stem–loop PCR. Plant *At*miR159a of 21 nt was used as a positive control. (B) Relative accumulation of the four amiRNAs in transformed Arabidopsis protoplasts at 24 hptr. The relative amounts of amir21, amir24, amir154, and amir296 were normalized to *At*miR159a. Values represent the mean ±SE of three technical replicates from one biological replicate.

Information on the amiRNAs is summarized in Supplementary Table S2, including their 5ʹ-terminal nucleotides, their predicted *At* target genes, their target aligned fragment, and their target prediction expectation values, indicating the degree of mismatches between the amiRNAs and the *At* target sequence. Alignment of the designed amiRNAs to their predicted target fragment is illustrated in Supplementary Fig. S3A and B. Subsequently, the four amiRNAs were cloned into the pUC18-*At*MIR390a-B/c-RFP vector and transformed into *At* protoplasts, reaching a transformation efficiency of 65–90% (calculated as the ratio between the red fluorescing protoplasts and the total number of viable protoplasts; Supplementary Fig. S6). The four amiRNAs were successfully expressed in protoplasts as detected by duplexed stem–loop PCR (Fig. 4A). Amplification products were cloned into the pGEM-T^®^ cloning vector, and sequencing confirmed amiRNAs sequences.

Next, we quantified amiRNA accumulation in protoplasts using stem–loop RT–qPCR. All four amiRNAs were successfully detected in transformed protoplasts, while they could not be found in non-transformed protoplasts, indicating a high accumulation (Fig. 4B).

**Fig. 5.**
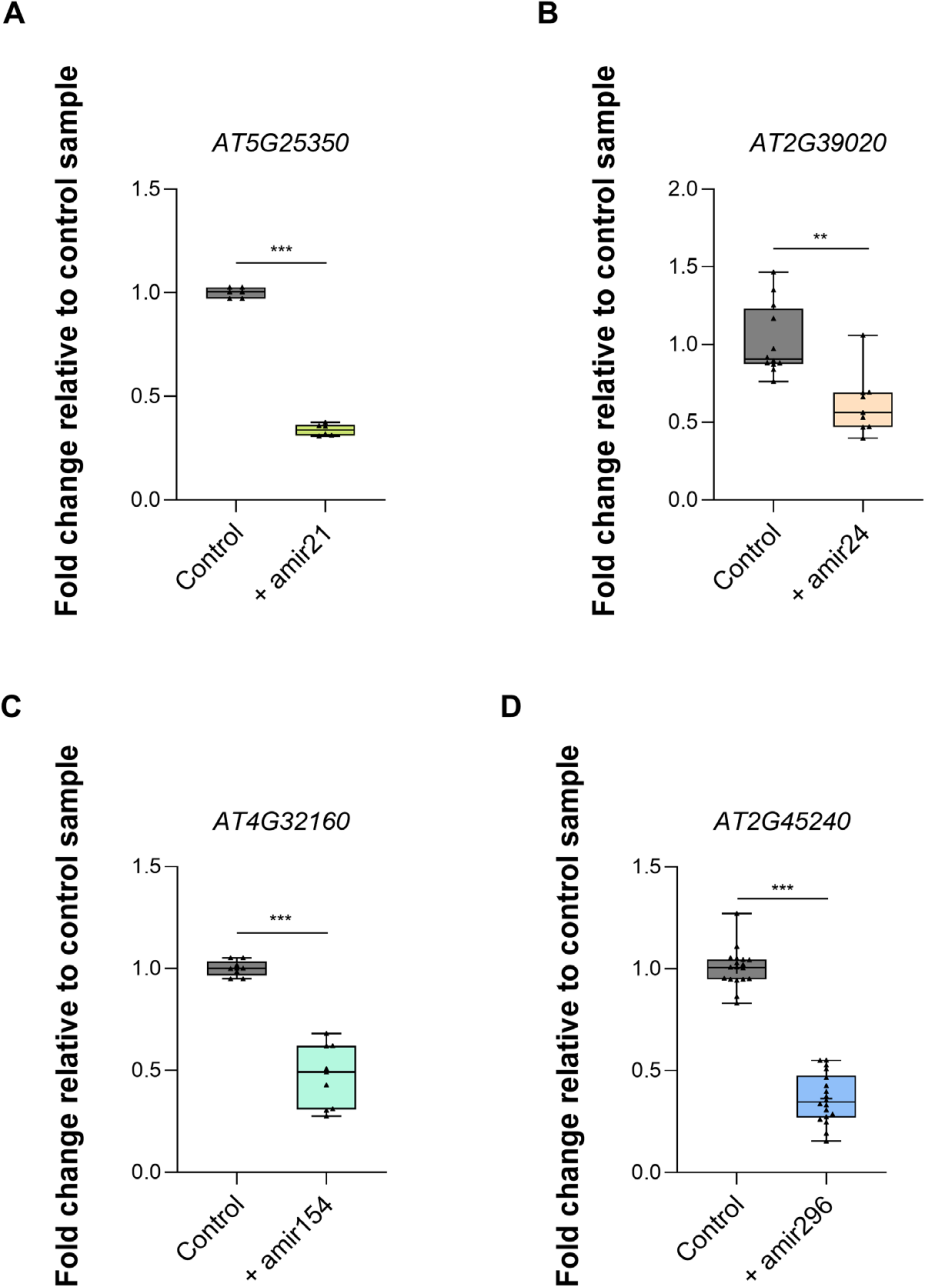
qPCR analysis of target gene silencing in Arabidopsis protoplasts 24 hptr with amiRNAs. The mRNA transcript levels of the target genes (A) *AT5G25350*, (B) *AT2G39020*, (C) *AT4G32160*, and (D) *AT2G45240* in transformed protoplasts (+) expressing amir21, amir24, amir154, or amir296 were normalized against the housekeeping gene *Ubiquitin* (*UBC21, AT5G25760*) and displayed as fold change relative to control protoplasts (without amiRNA construct). Data are the average of 2–3 biological replicates ±SD. Asterisks indicate a difference at *P≤0.05, **P≤0.01, and ***P≤0.001 according to Student’s *t*-test. ns=not significant.

Target gene down-regulation in protoplasts was further assessed by qPCR 24 hours post-transformation (hptr), revealing reductions of 68% for *AT5G25350*, 43% for *AT2G39020*, 50% for *AT4G32160*, and 65% for *AT2G4524*0 (Fig. 5).

### 5ʹ-RLM-RACE reveals canonical cleavage in protoplasts transformed with amiRNA

**Fig. 6.**
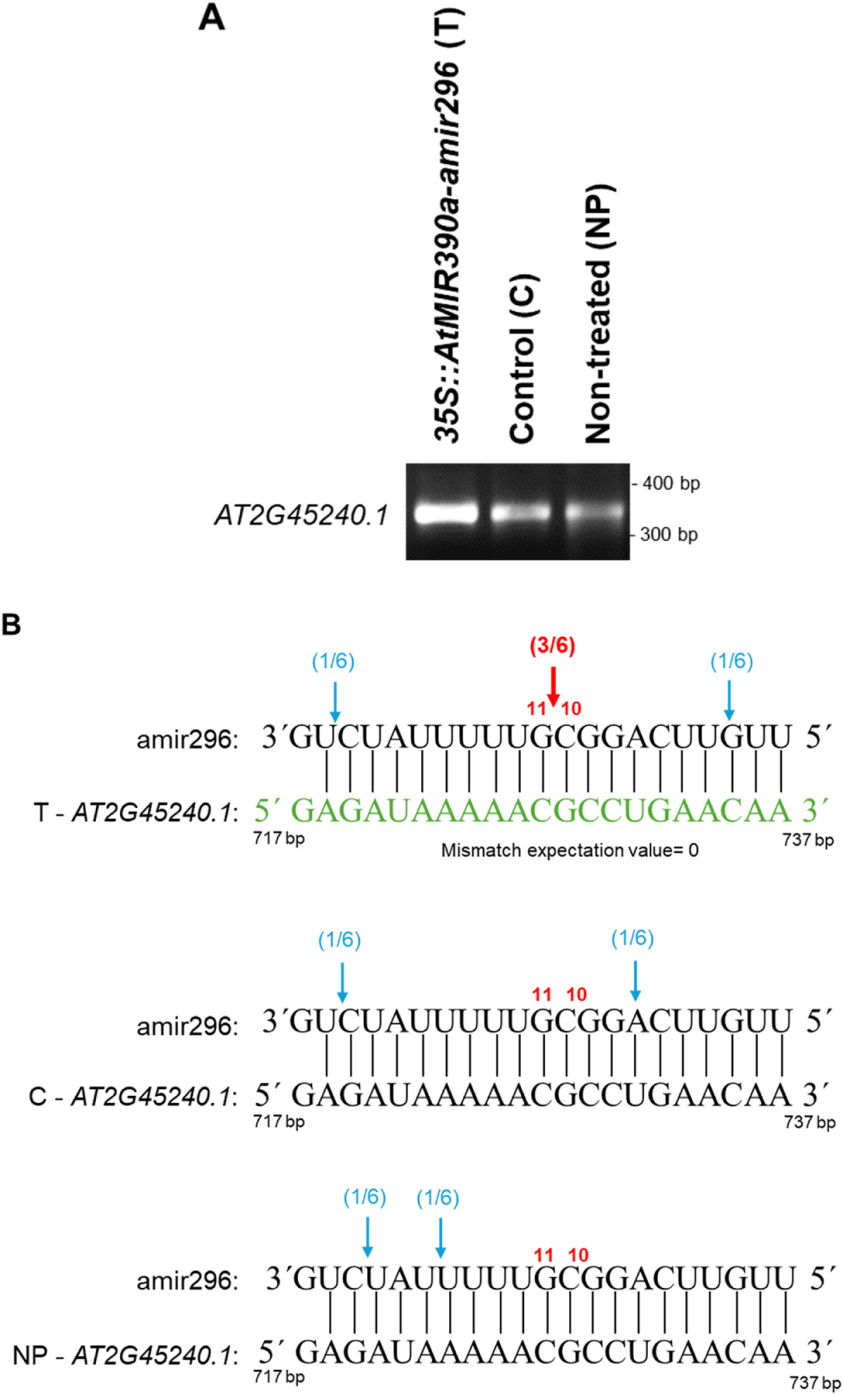
Identification of 5ʹ-RLM-RACE target sites in *AT2G45240* mRNA following amir296 expression in Arabidopsis protoplasts. (A) PCR products of the second nested 5ʹ-RLM-RACE-PCR visualized in an ethidium bromide–agarose gel. RNA was extracted from protoplasts transformed with amir296 (T) and from corresponding control protoplasts (C) and untreated protoplasts (NP). (B) Mapping of *AT2G45240* target cleavage products by 5ʹ-RLM-RACE. The predicted base pairing between amir296 and *AT2G45240* is shown in green. The red arrow indicates the detected canonical cleavage site, and the blue arrows indicate non-canonical cleavage occurring probably due to RNA degradation or a non-identified *At* miRNA action nearby. The proportion of cloned 5ʹ-RLM-RACE products at the different cleavage sites is shown in parentheses. For protoplasts transformed with amir296, three colonies have the 5ʹ end at the expected position, opposite to nucleotides 10–11 of amir296, which is not the case for control and untreated protoplasts. A mismatch expectation value (also called target predicted score) of 0 indicates an almost full complementarity between amir296 and the target gene *AT2G45240* (expectation values range between 0 and 5, with 0 being the highest level of sequence complementarity and 5 being the lowest).

We selected amir296 and its predicted target gene *AT2G45240* to investigate the canonical PTGS cleavage site by 5ʹ-RLM-RACE (Llave *et al.*, 2002; Ueno *et al.*, 2022). Samples were obtained from transformed protoplasts at 24 hptr, from control protoplasts (without amiRNA construct), and from non-treated protoplasts. A distinct band of ~383 bp was amplified from protoplasts expressing amir296, consistent with the expected size of the canonical cleavage product predicted to be targeted by amir296. Unexpectedly, weaker DNA fragments of a similar size were detected in both control and non-treated protoplasts (Fig. 6A). Cloning and subsequent sequencing of the amplicons confirmed that all sequences aligned with the *AT2G45240* sequence, and 50% of the obtained sequences from protoplasts transformed with pUC18-*At*MIR390a-RFP-amir296 (three of six clones) exhibited the canonical cleavage site between nucleotides 10 and 11 of amir296. In contrast, PCR products from control and non-treated protoplasts contained an *AT2G45240* fragment, but did not correspond to a canonical cleavage site, suggesting a possible cleavage by an endogenous miRNA near the amir296-binding site or RNA degradation (Fig. 6B).

### Effector-like *Si*sRNA candidates mediate PTGS in transformed Arabidopsis protoplasts

**Fig. 7.**
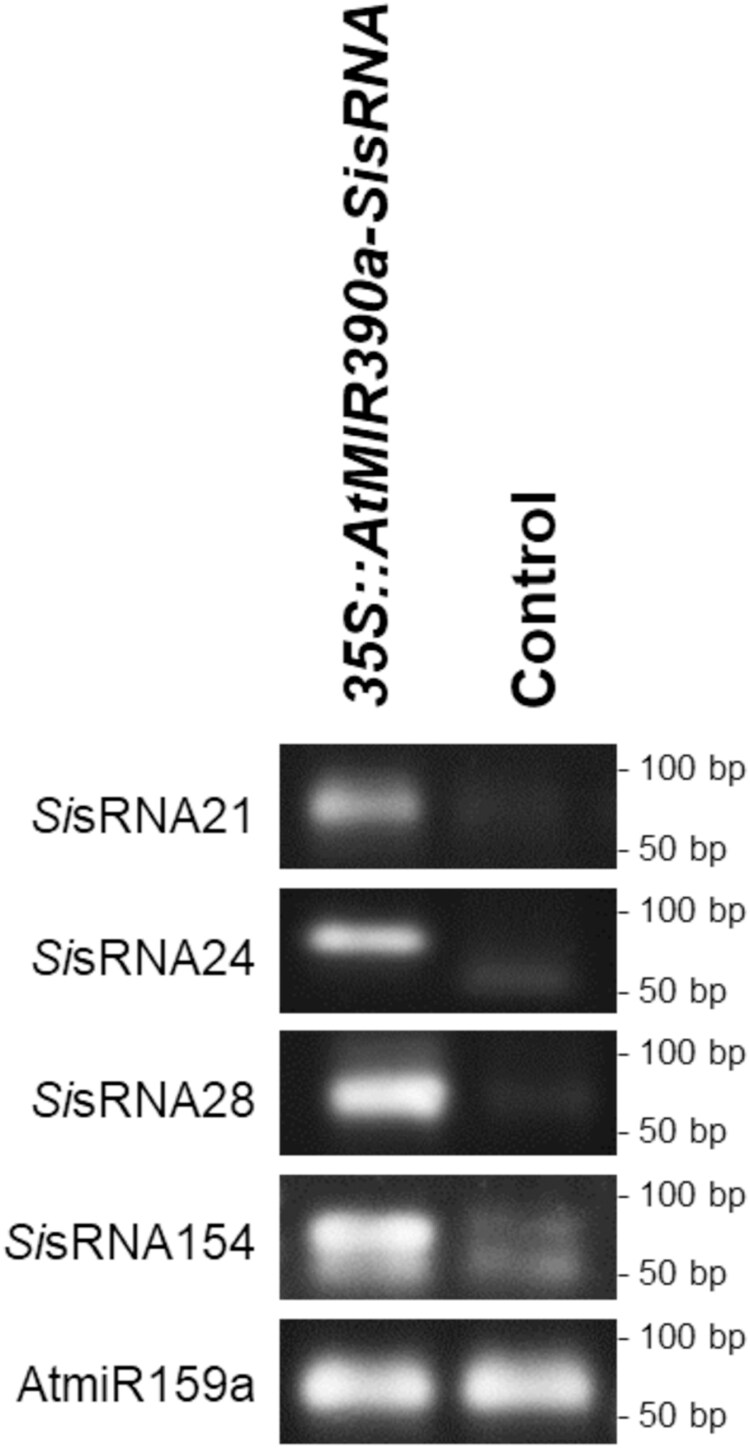
Duplexed stem–loop PCR confirming expression of *Si*sRNAs in Arabidopsis protoplasts. Agarose gel analysis of stem–loop PCR detected expression of all four *Si*sRNAs in transformed protoplasts at 24 hptr, but not in control protoplasts (without *Si*sRNA construct). No amplification or weak bands of primer dimers were seen in the control protoplasts (without construct) samples, probably due to the high number of cycles in the stem–loop PCR. Plant *At*miR159a of 21 nt was used as a positive control.

Next, we transiently transformed protoplasts with *Si*sRNAs to demonstrate their gene silencing activity. *At* protoplasts were transformed with the pUC18-*At*MIR390a-RFP expression construct containing *Si*sRNA21, *Si*sRNA24, *Si*sRNA28, and *Si*sRNA154, respectively (confirmed in axenic culture and *Si–At* interaction). Duplexed stem–loop PCR confirmed the expression and amplification of the four *Si*sRNAs (Fig. 7). The amplification products were cloned into the pGEM-T^®^ cloning vector, and sequencing confirmed their identity. Validation of *Si*sRNA accumulation was performed using stem–loop RT–qPCR for *Si*sRNA28 and *Si*sRNA154 to further confirm the success of the protoplast transformation system (Supplementary Fig. S7).

**Fig. 8.**
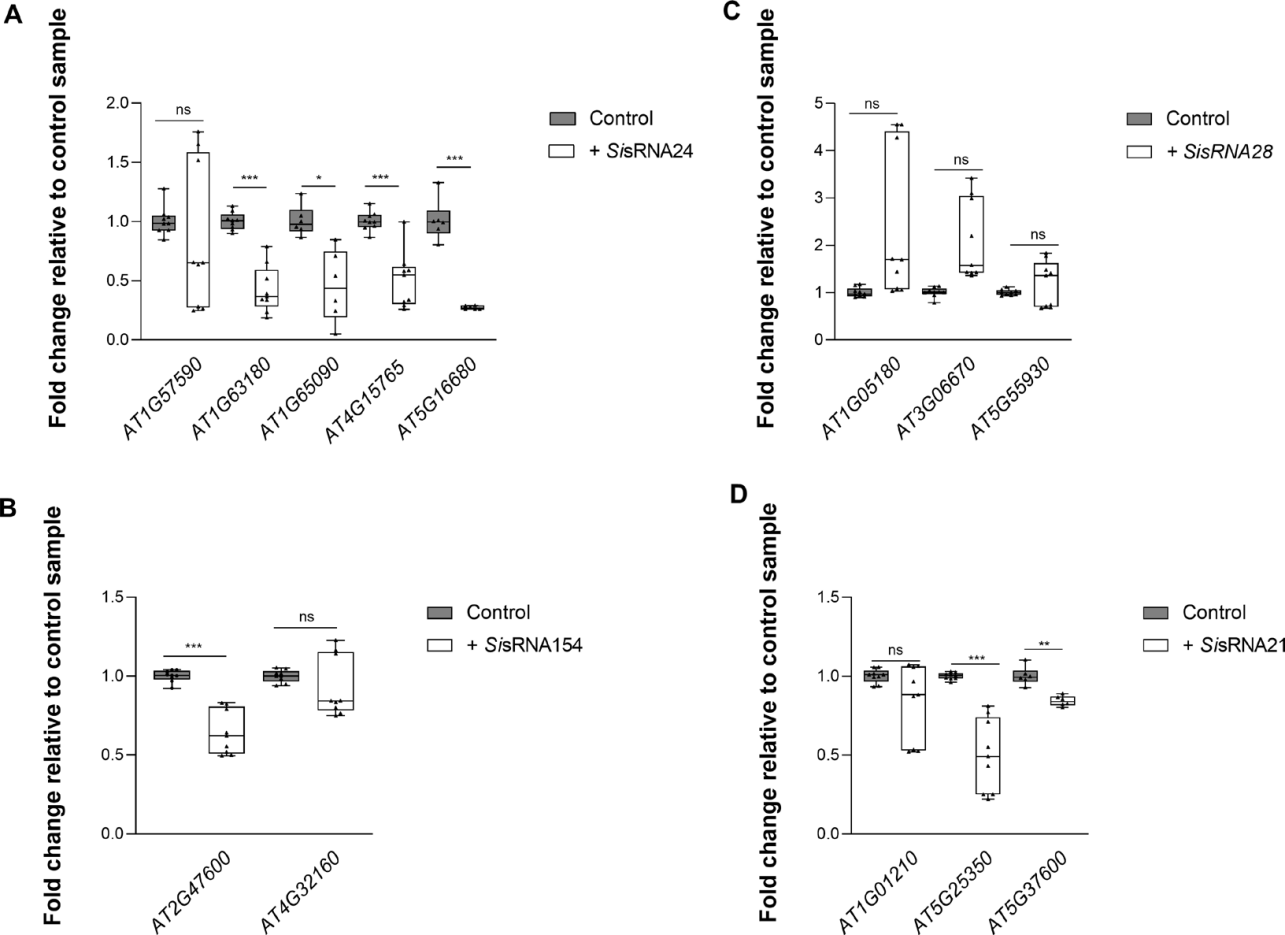
qPCR analysis of target gene silencing in Arabidopsis protoplasts 24 hptr with *Si*sRNAs versus control protoplasts (without *Si*sRNA construct). The mRNA transcript levels of target genes in transformed protoplasts (+) expressing (A) *Si*sRNA24, (B) *Si*sRNA154, (C) *Si*sRNA28, and (D) *Si*sRNA21 normalized against the housekeeping gene *Ubiquitin* (*UBC21*, *AT5G25760*) are displayed as fold change relative to control protoplasts (without *Si*sRNA construct). Data are the average of 2–3 biological replicates ±SD. Asterisks indicate a difference at *P≤0.05, **P≤0.01, and ***P≤0.001 according to Student’s *t*-test. ns=not significant.

Next, we investigated the ability of the expressed *Si*sRNAs to induce PTGS of predicted target genes in protoplasts. From 199 Arabidopsis target genes of *Si*sRNA21, *Si*sRNA24, *Si*sRNA28, and *Si*sRNA154 predicted using psRNATarget (Supplementary Dataset S1), we selected 13 genes for further analysis, prioritizing those with intriguing descriptions and with a potential involvement in mutualistic interaction and plant immunity. *Si*sRNA24 was predicted to target five *At* genes: *AT1G57590* (*PECTIN ACETYLESTERASE 2*), involved in cell wall organization, *AT1G63180* (*UDP-**d**-GALACTOSE 4-EPIMERASE 3*), involved in pollen development, *AT1G65090* (*SEED LIPID DROPLET PROTEIN1*), involved in lipid droplet–plasma membrane tethering, *AT4G15765* [*FAD/NAD*(*P*)*-BINDING OXIDOREDUCTASE FAMILY PROTEIN*], involved in jasmonic acid-mediated signalling, and *AT5G16680* (*Protein PARALOG OF AIPP2, PAIPP2, PHD2*), involved in regulation of gene expression (Supplementary Dataset S1; Supplementary Table S3). Expression of *Si*sRNA24, which carries a U at the 5ʹ-terminus, and thus a preference for AGO1 (Mi *et al.*, 2008; Montgomery *et al.*, 2008; Takeda *et al.*, 2008), significantly reduced the mRNA abundance of *AT1G63180*, *AT1G65090*, *AT4G15765*, and *AT5G16680* in protoplasts 24 hptr by 63, 56, 45, and 73%, respectively (Fig. 8A). Similarly, *Si*sRNA154, also with 5ʹ-U, is predicted to target *AT2G47600* (*MAGNESIUM/PROTON EXCHANGER*) involved in magnesium, iron, and zinc ion transport, and *AT4G32160* [*Phox* (*PX*) *DOMAIN-CONTAINING PROTEIN EREL1*], involved in signal transduction. Expression of *Si*sRNA154 significantly down-regulated the accumulation of *AT2G47600* mRNA by 37%, but not of *AT4G32160* (Fig. 8B). *Si*sRNA28 has a 5ʹ-A, therefore a loading preference for AGO2 and AGO4 (Mi *et al.*, 2008; Montgomery *et al.*, 2008; Takeda *et al.*, 2008). Expression of *Si*sRNA28 in protoplasts did not result in down-regulation of any of the three predicted target genes, namely *AT1G05180* (*AUXIN RESISTANT 1*), involved in auxin-activated signalling pathway and response to cytokinin, *AT5G55930* (*ARABIDOPSIS THALIANA OLIGOPEPTIDE TRANSPORTER 1*), an oligopeptide transporter, and *AT3G06670* (*PLATINUM SENSITIVE 2 LIKE*) with a regulatory function in non-coding RNA processing (Fig. 8C). Finally, expression of *Si*sRNA21 with 5ʹ-C and thus a preference for AGO5 (Mi *et al.*, 2008; Montgomery *et al.*, 2008; Takeda *et al.*, 2008) resulted in down-regulation of predicted target transcripts *AT5G25350* (*EIN3-BINDING F BOX PROTEIN 2*), a negative regulator of the ethylene-activated signalling pathway, and of *AT5G37600* (*ARABIDOPSIS GLUTAMINE SYNTHASE 1*), involved in nitrate assimilation, by 51% and 16%, respectively (Fig. 8D).

### 5ʹ-RLM-RACE reveals non-canonical cleavage of Arabidopsis target genes in protoplasts expressing *Si*sRNAs with preference for AGO1

We selected *Si*sRNA24 to study the cleavage pattern of the down-regulated target transcripts *AT1G65090* and *AT4G15765* using 5ʹ-RLM-RACE. For the predicted target gene *AT1G65090*, no band corresponding to an expected canonical cleavage product was detected. For the target gene *AT4G15765*, we detected a single band of 300 bp in protoplasts expressing *Si*sRNA24, which was shorter than the expected size (443 bp), while double bands were observed for the control protoplasts (Supplementary Fig. S8A). PCR amplicons from both transformed and control protoplasts were cloned into the pGEM-T^®^ cloning vector and sequenced. Sequence analysis confirmed the alignment of the 300 bp RLM-RACE product with *AT4G15765*. However, it did not confirm the 5ʹ end position, between nucleotides 10 and 11, predicted for *Sis*RNA24-guided canonical cleavage (Supplementary Fig. S8B). The absence of cleavage and/or a canonical cleavage product does not exclude the potential of *Si*sRNA24 in mediating predicted *At* gene silencing, as many previous studies have highlighted the challenge of identifying sRNA cleavage sites in plants (Brousse *et al.*, 2014; Werner *et al.*, 2021).

### *Si–At* interaction induces changes in predicted Arabidopsis target genes

**Fig. 9.**
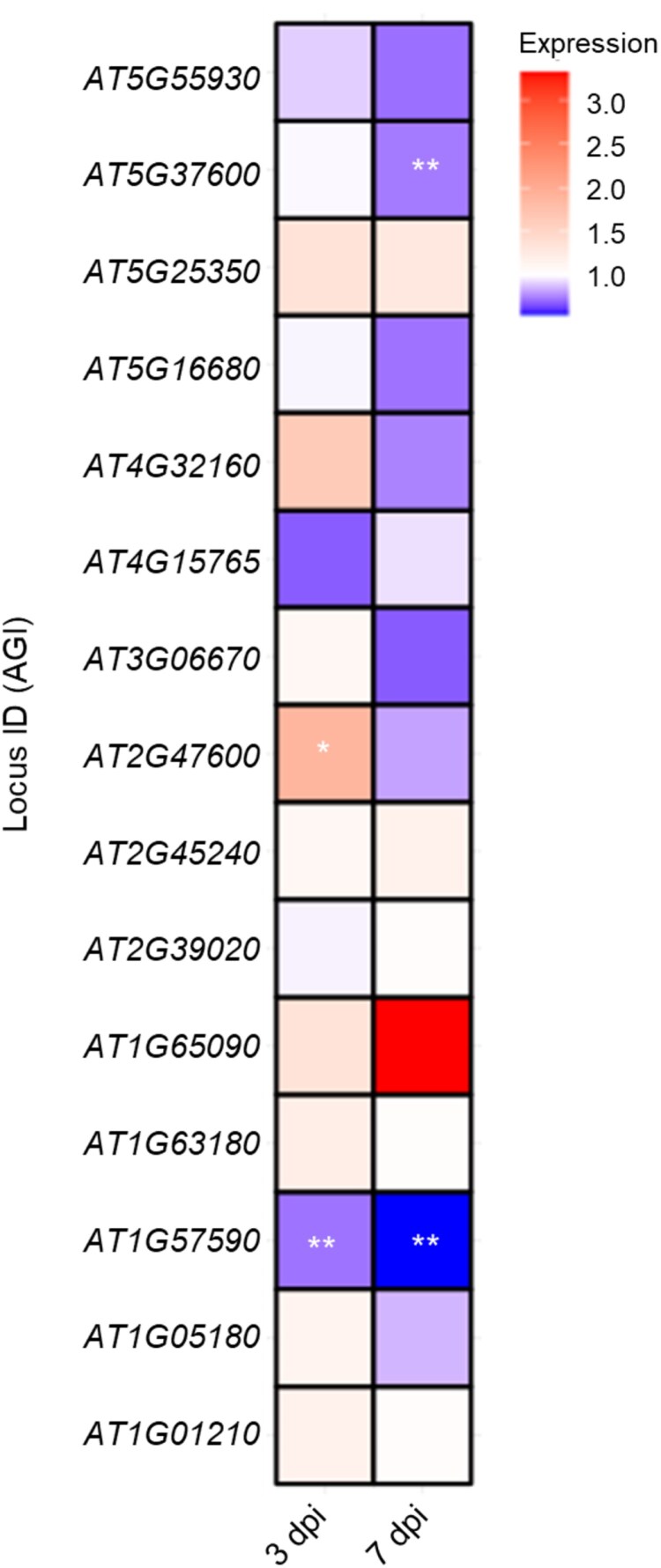
Expression analysis of predicted Arabidopsis target genes under *Serendipita indica* colonization. The heat map shows transcript abundance as fold change of *At* genes assessed at 3 and 7 days post-inoculation (dpi) of root colonization. Colour intensity within each cell corresponds to the change in gene expression relative to non-inoculated samples, with red indicating up-regulation and blue indicating down-regulation. Data represent the mean of two independent biological replicates. Asterisks indicate significant differences at *P≤0.05, **P≤0.01, and ***P≤0.001 based on Student’s *t*-test.

To assess how the symbiotic interaction modulates gene expression in *At* roots, we examined the expression levels of the 15 predicted *At* target genes analysed for silencing in protoplasts transformed with amiRNAs and *Si*sRNAs. We performed RT–qPCR on inoculated and mock-treated (non-inoculated) *At* roots at 3 and 7 dpi, and found dynamic, time-dependent changes in gene expression, with distinct expression patterns observed at 3 and 7 dpi. A general trend of down-regulation was observed at 7 dpi for the majority of the target genes analysed. The differential expression patterns in the *Si*–*At* interaction are shown in Fig. 9.

### Effector-like *Si*sRNA candidates are loaded onto Arabidopsis AGO1

**Fig. 10.**
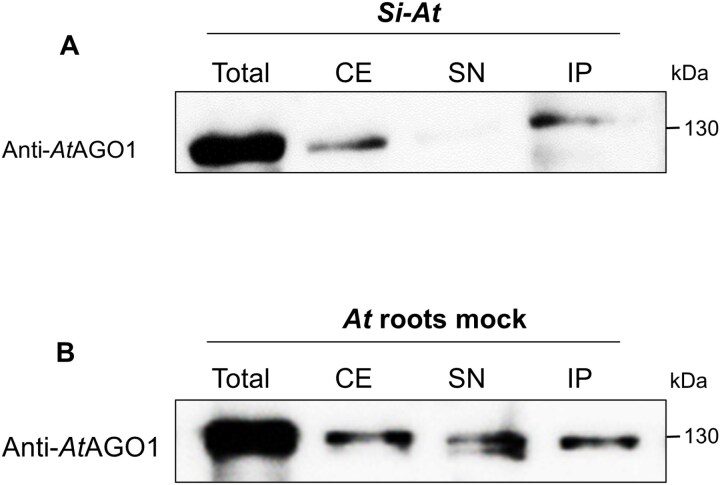
Quality control of AGO1 co-immunoprecipitation by western blot analysis. Four sample fractions of the AGO1 Co-IP experiment were analysed: total protein before centrifugation and debris removal; crude extract (CE) after centrifugation and debris removal; supernatant (SN) after incubation with anti-*At*AGO1 and agarose beads; and IP fraction (resuspended IP pellet). The four fractions were analysed in *Si*-colonized roots (A) and mock-treated *At* roots (B). Both panels show the detection of *At*AGO1 using anti-*At*AGO1-specific antibody at the expected size of ~130 kDa. Of note, AGO1 signals were stronger in IP fractions than in SN. The broad-range pre-stained protein marker was used as a protein size marker.

To further substantiate the hypothesis that *Si*sRNAs are loaded into the *At* RISC during the *Si–At* interaction, we conducted *At*AGO1–sRNA Co-IP using samples of *Si*-colonized roots, followed by stem–loop PCR analysis (Carbonell, 2017b; Dunker *et al.*, 2021). To exclude the possibility that the *At*-specific anti-AGO1 antibody would cross-react with fungal AGOs, we first performed an *At*AGO1–sRNA Co-IP followed by western blot analysis using a 4-week-old axenic culture. No binding of the *At*AGO1-specific antibody was detected in the crude extract either in the supernatant or in the IP fraction in comparison with the total loaded proteins from *Si* axenic culture, confirming the specificity of the plant anti-AGO1 antibody (Supplementary Fig. S9A). In line with this, no *Si*sRNAs could be detected by stem–loop PCR using *Si*sRNA-specific stem–loop primers (Supplementary Fig. S9B). Similarly, western blot analysis of *At*AGO1-IP of samples from *Si*-colonized *At* roots and *At* mock-treated roots was performed. AGO1 accumulation was observed in crude extracts and IP fractions, with a stronger signal in the IP fraction than in the supernatant from the *Si–At* sample (Fig. 10A, B).

**Fig. 11.**
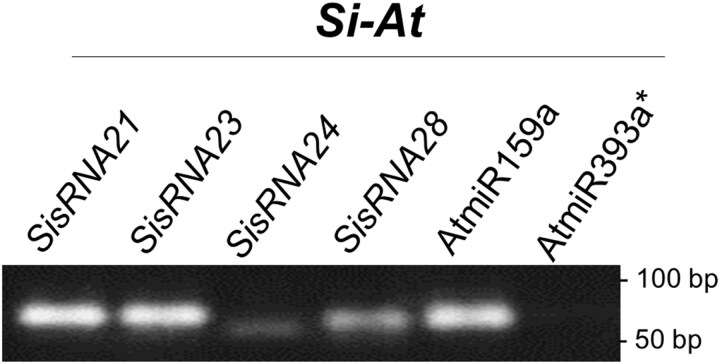
*Si*sRNAs co-immunopurified with *At*AGO1 in samples from *Si*-colonized Arabidopsis roots. Stem–loop PCR confirms the presence of *Si*sRNA21, *Si*sRNA23, *Si*sRNA24, and *Si*sRNA28, as well as AGO1-bound *At*miR159a, but not AGO2-bound *At*miR393a* in AGO1 Co-IP samples from *Si*-colonized *At* roots harvested at 7 days post-inoculation.

Next, we recovered the *At*AGO1-bound sRNA from the Co-IP sample of *Si*-colonized roots (*Si–At*). Stem–loop PCR was performed and detected the *At*AGO1-bound *At*miR159a and a band of the expected size for *Si*sRNA21, *Si*sRNA23, *Si*sRNA24, and *Si*sRNA28 (Fig. 11), whereas *At*miR393a*, which is known to preferably bind *At*AGO2 (Mi *et al.*, 2008), was undetectable. Stem–loop PCR products from the *Si–At* Co-IP fraction were cloned and sequenced. The sequencing results confirmed the presence of plant *At*miR159a as well as *Si*sRNA24 and *Si*sRNA28, with the exact same sequences detected from axenic culture and *Si*-colonized *At* roots, confirming the success of the pull-down. In addition, two *Si*sRNAs with almost identical sequences to *Si*sRNA21 and *Si*sRNA23 were also co-immunoprecipitated (Supplementary Table S9). Such mismatches may be caused by the processing steps of the sRNAs until the loading into the AGO1, which may result in sequence variations compared with the total RNAseq performed in our previous work (Šečić *et al.*, 2021). Nevertheless, these results show that detected *Si*sRNAs are bound to *At*AGO1, supporting the notion that *Si*sRNAs are transferred into *At* root cells during root colonization and loaded into the plant RNAi machinery.

A systematic overview of the investigated *Si*sRNAs and their experimental results is provided in Supplementary Table S10. In conclusion, this study provides a blueprint for rapid selection and analysis of sRNA effectors and further supports the model of cross-kingdom communication in Sebacinalean symbiosis.

## Discussion

In this work, we developed an experimental pipeline (Fig. 1) for the analysis of putative fungal sRNA effectors and investigated their role in host target gene silencing. We have previously reported the identification and analysis of fungal *Si*sRNAs induced in the interaction of the beneficial root endophyte *S. indica* with *B. distachyon* (Šečić *et al.*, 2021). To bridge the gap between the large amount of bioinformatic data available from this previous study and the functional validation of the *Si*sRNAs, we established a rapid and reproducible roadmap for the validation of *Si*sRNAs using an Arabidopsis protoplast system. First, we validated the effectiveness of our pipeline by expressing amiRNAs and *Si*sRNA in protoplasts and confirmed their accumulation and their potential to induce PTGS of predicted *At* target genes. Moreover, we confirmed the accumulation of the selected *Si*sRNAs in *Si*-colonized roots, where fungal colonization induced transcriptional changes in some of the investigated *At* genes. Second, using IP pull-down assay, we showed that *Si*sRNAs are loaded into the plant RNAi machinery, suggesting the translocation of *Si*sRNAs into host cells. Following that, we detected *Si*sRNA24 and *Si*sRNA28 bound to *At*AGO1, supporting the ckRNAi hypothesis.

To test the functionality of the selected *Si*sRNAs in the Sebacinoid symbiosis, we generated a construct to express sRNAs in *At* protoplasts based on the pUC18 backbone vector and the *At*MIR390a distal stem–loop, which can generate 21 nt amiRNAs or *Si*sRNAs. The *At*MIR390a precursor belongs to the MIR390 family, which is highly conserved and precisely produced and processed in several plant species (Axtell *et al.*, 2006; Cuperus *et al.*, 2011). Carbonell *et al.* (2014) tested the functionality of the *At*MIR390a-based amiRNA vector in repressing target gene accumulation in *At* plants. Using the plant vector pMDC32B-*At*MIR390a-B/c enables the directed cloning of amiRNAs and their stable expression in dicotyledonous plants. Although MIR390 associates preferably with AGO7, the association of *At*MIR390a-derived amiRNAs that have a 5ʹ-U can be directed to AGO1 (Montgomery *et al.*, 2008; Cuperus *et al.*, 2010; Carbonell *et al.*, 2014; Carbonell, 2017b). Conventionally, sRNA–AGO1-mediated cleavage results in a canonical cut between nucleotides 10 and 11 of the sRNA at the corresponding mRNA site. This cleavage is a confirmation of sRNA loading into AGO1, formation of the RISC, and the processing of the mRNA transcript (Addo-Quaye *et al.*, 2009; Huntzinger and Izaurralde, 2011). In our approach, we combined the advantages of plant expression vectors and the protoplast transient expression system, both considered valuable tools, to study gene silencing and the molecular mechanisms of RNAi in plants (Tyurin *et al.*, 2020). *At* protoplasts offer some significant advantages for studying and validating sRNA functionalities. As *At* is a well-characterized model organism with a well-characterized RNAi machinery, *At* protoplasts enable detailed mechanistic studies of sRNA processing and AGO loading. Moreover, using *At* protoplasts allows the study of the *Si*sRNA–*At* predicted target interaction within a native biological and a genetically compatible context as compared with alternative—often used—transient transformation systems such as, for example, *Nicotiana benthamiana* protoplasts, which would require heterologous expression of both *Si*sRNAs and their predicted targets, thus complicating experiments and introducing new variabilities. While the most appropriate transformation system would be the use of *At* root protoplasts, their isolation is challenging due to the complexity of root cell walls, resulting in low cell yield and viability (Pasternak *et al.*, 2021). Moreover, root protoplasting results in a mixture of protoplasts derived from different cell types, while *Si* only colonizes rhizodermis and cortex cells (Jacobs *et al.*, 2011). Overall the *At* protoplast system offers better regulatory control and biological relevance, particularly for exploring root symbiosis, as demonstrated in previous studies involving *S. indica* (Osborne *et al.*, 2023).

To confirm the robustness of the protoplast transformation system, we first used amiRNAs with an almost perfect match to a predicted plant target gene. When expressed in *At* protoplasts, all four amiRNAs, namely amir21, amir24, amir154, and amir296 (Supplementary Table S2), induced a significant reduction in abundance of their corresponding target genes (Fig. 5), confirming their silencing activity in the protoplast system. amiRNAs used in our pipeline are algorithmically designed to exhibit a 5ʹ-U, thereby enabling their loading into the AGO1 protein (Carbonell, 2017c). Consequently, they can enter the RISC and induce a down-regulation of the target gene. Accordingly, amir296, which mediated a 65% reduction of its predicted target *AT2G45240* (*METHIONINE AMINOPEPTIDASE 1A, MAP1A*), can direct a canonical cleavage, as detected by 5ʹ-RLM-RACE between position 10 and 11 of amir296, confirming an *At*AGO1-mediated slicing of *AT2G45240* mRNA (Fig. 6).

We also confirmed by stem–loop PCR the expression and accumulation of the putative *Si*sRNA24 effector, exhibiting a 5ʹ-U. Four of five predicted targets of *Si*sRNA24 showed a significantly reduced abundance in the transformed protoplasts (Fig. 8A). Moreover, *Si*sRNA154, which also has a 5ʹ-U, directed down-regulation of *AT2G47600*, one of the two *At* predicted target genes (Fig. 8B).

An important factor to consider when studying PTGS mediated by putative sRNA effectors is the degree of mismatch. Expressed as ‘Expectation value’ or ‘Target predicted score’, it reports on the mismatch score between the predicted target mRNA and the sRNA. Previous studies have demonstrated that the number and position of mismatches between the sRNA and the corresponding target mRNA can significantly impact their binding affinity, eventually weakening the interaction and resulting in either non-significant or no down-regulation (Rhoades *et al.*, 2002; Ossowski *et al.*, 2008; Carbonell *et al.*, 2015). Studies have shown that mismatches within the sRNA seed region spanning nucleotides 2–12/13 are possible, but they may reduce the sRNA activity, whereas mismatches at position 1 or between nucleotides 14 and 21 are more tolerable (Mallory *et al.*, 2004; Carbonell, 2017a). Moreover, mismatches can also lead to a different mode of sRNA action. A mismatch can trigger a deadenylation pathway or a translational repression pathway instead of an mRNA degradation pathway, leading to a decrease in protein synthesis instead of mRNA degradation (Baulcombe, 2004; Carthew and Sontheimer, 2009). We hypothesize that a high abundance level of a target mRNA may lead to less detectable down-regulation, as its accessibility for the sRNA may have an impact on the silencing process and the detection of a canonical cleavage. It is also conceivable that the presence of other endogenous miRNAs, which may bind near or at the same mRNA-binding site, might disrupt sRNA–mRNA binding, modulate the regulatory outcome, and affect the gene expression pattern.

Further analysis of the molecular cleavage pattern of *Si*sRNA24 by 5ʹ-RLM-RACE identified non-canonical cut sites in the target gene *AT4G15765.* This finding may be explained by the low abundance of the cleaved product, falling below the detection threshold of the RLM-RACE method. This low abundance would make the detection of a precise canonical cleavage challenging. Alternatively, it might also be possible that the cleavage site within the target mRNA is located at a different position. Hence, non-canonical cleavage sites of sRNA may be further investigated as several studies speculate that PTGS can still be induced through sRNA or phasiRNA directing non-canonical cleavage (Brousse *et al.*, 2014; Ivanova *et al.*, 2022). High-throughput sequencing of samples from the *Si–At* interaction, including transcriptional analysis and degradome sequencing, is needed to better understand *Si*sRNA-mediated target cleavage.

In our study, the Arabidopsis target *AT5G25350* is predicted to be targeted by both amir21 and *Si*sRNA21. When expressing amir21 (with 5ʹ-U and an expectation value of 0), the mRNA abundance of *AT5G25350* declined to 68% (Fig. 5A). However, when expressing *Si*sRNA21 (with a 5ʹ-C and an expectation value of 5), we observed down-regulation of 51% of the same target gene (Fig. 8D). Another example is the predicted gene *AT4G32160* targeted by amir154 and *Si*sRNA154, both with a 5ʹ-U. The mRNA abundance of *AT4G32160* in protoplasts expressing amir154 (with an expectation value of 0) resulted in a reduction of 50% (Fig. 5C), but protoplasts expressing *Si*sRNA154 (with an expectation value of 4.5) did not show a reduction of *AT4G32160* transcript (Fig. 8B; overview in Supplementary Fig. S10). These results raise the possibility that amiRNAs are more prone to mediate target gene silencing, but further studies with additional amiRNAs and putative sRNAs are required to confirm these observations.

Analysis of host gene expression in response to *Si* colonization, together with PTGS induced by *Si*sRNAs in protoplasts, revealed significant patterns. *AT1G57590* encodes a pectin acetyl esterase (*PAE2*) that modifies pectin in plant cell walls, affecting their rigidity, porosity, and interactions with microbes. The gene was significantly down-regulated in *At* roots upon *Si* colonization at 3 and 7 dpi (Fig. 9). This finding suggests that the fungus manipulates cell wall properties to facilitate its colonization by increasing cell wall permeability. Single-cell RNA sequencing of *At* root showed that *PAE2* is highly expressed in the root rhizodermis (Rich-Griffin *et al.*, 2020), the primary tissue colonized by *Si* (Jacobs *et al.*, 2011). Interestingly, and in clear contrast, *PAE2* is up-regulated in *At* Col-0 leaves during infection by the necrotrophic pathogen *Botrytis cinerea* (Windram *et al.*, 2012) and by biotrophic *Pseudomonas syringae* pv. tomato DC3000 (Zhang *et al.*, 2007), suggesting an ambivalent role in plant–microbe interactions and in plant defence.

Although *Si*sRNA24 targets *PAE2*, no down-regulation was observed in transformed protoplasts (Fig. 8A), which could be attributed to the low expression of *PAE2* in leaves (Schmid *et al.*, 2005) or to the importance of tissue-specific signalling in plant–mutual interactions, warranting further investigation.

Two other interesting genes are *AT5G37600*, encoding GLUTAMINE SYNTHASE 1 (GS1), a cytosolic enzyme involved in nitrogen assimilation by converting ammonium to glutamine, and *AT2G47600* encoding MAGNESIUM/PROTON EXCHANGER (*ATMHX*). Nitrogen metabolism and magnesium and iron transport are crucial for plant growth and the uptake of essential nutrients by the plant. Both proteins are associated with immune responses, particularly in the regulation of mutualistic interactions. *GS1* was down-regulated at 7 dpi upon *Si* colonization (Fig. 9), suggesting that the plant is potentially modulating its nitrogen assimilation pathways in response to the fungal endophyte*. ATMHX* was also down-regulated at 7 dpi upon *Si* colonization (Fig. 9). Overall, the reduced nitrogen assimilation and magnesium/iron transport activity may reflect a shift in resource allocation that reduces nitrogen uptake and manipulates the nutrient transport to favour *Si* colonization. Additionally, or alternatively, the down-regulation of these genes suggests that *Si* modifies related signalling pathways. This supports the hypothesis presented by Scholz *et al.* (2023), suggesting that *Si* supports nitrogen-starved *At* seedlings by supplying nitrogen metabolites, thereby moderating metabolic nitrogen deficiency responses and reprogramming the expression of nitrogen metabolism-related genes. Of note, both *GS1* and *ATMHX* were confirmed to be down-regulated in protoplasts transformed with *Si*sRNA21 and *Si*sRNA24. In contrast, they are up-regulated in response to infection by *B. cinerea* and *P. syringae* (Zhang *et al.*, 2007; Windram *et al.*, 2012), suggesting a broader role in plant immune responses and mutualism. Moreover, both *GS1* and *ATMHX* are highly expressed in mock-treated *At* leaves and roots, particularly in the pericycle, cortex, and rhizodermis (Schmid *et al.*, 2005; Rich-Griffin *et al.*, 2020). Overall, these previous reports along with our findings strongly suggest the hypothesis that *Si* uses sRNAs to manipulate host nitrogen metabolism. Together, the data suggest that down-regulation of specific Arabidopsis genes during the Sebacinalean symbiosis does not seem to be a random event, but an effective mechanism by which the fungus manipulates the fitness of the host plant. By silencing host genes, *Si* creates a favourable environment for its colonization and nutrient acquisition, a phenomenon well documented in the literature (Qiang *et al.*, 2012; Hua *et al.*, 2017).

Hence, our data are consistent with the model that ckRNAi has evolved as an efficient mechanism in symbiosis of a plant and a fungus, striking a balance between host resistance and symbiotic benefits. Further investigation into these processes will provide deeper insights into the evolutionary advantages of ckRNAi in plant–microbe interactions.

We further evaluated ckRNAi by *At*AGO1–sRNA Co-IP on samples from *Si*-colonized *At* roots. Western blot analysis showed the enrichment of the *At*AGO1 in the IP fraction (Fig. 10). *Si*sRNA24 and *Si*sRNA28 were subsequently shown by stem–loop PCR to co-precipitate with *At*AGO1 (Fig. 11), providing strong evidence for the translocation of *Si*sRNAs and their incorporation into the RNAi machinery of *At*. Importantly, the identification in the *At*AGO1-IP of two *Si*sRNAs exhibiting nucleotide variations at their 3ʹ terminus to the previously identified *Si*sRNA (*Si*sRNA21 and *Si*sRNA23) suggests alternative *Si*sRNA variants. The presence of such variants highlights the complexity of the sRNA-mediated regulatory networks during *Si*–*At* root colonization. AGO-IP is a straightforward method to experimentally validate the loading of sRNAs into AGO proteins and to identify biologically functional sRNAs and miRNAs. However, the possibility of post-cell lysis association of sRNAs with AGOs cannot be excluded. Although testing for sRNA binding during sample lysis is not a common practice in AGO-IP workflows, it can rule out post-lysis AGO1–sRNA artifacts, as shown in Brosnan *et al*. (2019). Importantly, the absence of *At*miR393a*—preferentially loaded into AGO2—in our AGO1–sRNA Co-IP further supports that the detected AGO1-specific miRNAs and *Si*sRNAs were loaded *in vivo*. Moreover, independent confirmation of sRNA–mRNA binding and target down-regulation, as used in our pipeline, strengthens the evidence that *Si*sRNAs are incorporated into the RISC, rather than being the result of post-lysis artifacts.

In conclusion, we have developed a transient protoplast expression system to investigate the potential role of putative effector-like *Si*sRNA candidates in regulating target gene expression in *At*. This validation tool will not only help to understand the underlying molecular mechanism of *Si*sRNA-mediated PTGS but will also allow us to examine ckRNAi in mutualistic plant–fungal association. By studying *Si*sRNA-mediated PTGS, we have gained first insights that the endophytic fungus *Si* uses effector-like sRNAs to establish its mutualistic symbiosis with plants by modulating nitrogen metabolism and magnesium and iron transport as one strategy. Further studies will show whether *Si* utilizes its sRNAs to modulate the plant immune system, and additional genes may be identified as differentially regulated in symbiotic versus parasitic interactions.

Finally, amiRNAs are effective tools for fine-tuning gene expression in plants with a high silencing efficacy and thus are especially interesting for RNAi-mediated crop improvement strategies.

## Supplementary data

The following supplementary data are available at *JXB* online.

Table S1. Information summary of the 14 *Si*sRNAs selected for this study.

Table S2. Sequences of amiRNAs cloned in the pUC18-*At*MIR390a-B/c-RFP construct and expressed in *At* protoplasts for PTGS validation.

Table S3. Sequences of *Si*sRNAs cloned in the pUC18-*At*MIR390a-B/c-RFP construct and expressed in *At* protoplasts for PTGS validation.

Table S4. Seventy five-mer oligonucleotides used for cloning *Si*sRNAs and amiRNAs into the pUC18-AtMIR390a-RFP vector.

Table S5. Primers used in stem–loop end-point and stem–loop qPCR.

Table S6. Primers used for gene expression analysis in qPCR.

Table S7. Primers used for RLM-RACE.

Table S8. PCR primers.

Table S9. Sequences of *Si*sRNAs detected in *Si* axenic and *Si–At* interaction compared with the respective stem–loop PCR amplicons detected from *Si–At* Co-IP.

Table S10. Summary of the selected *Si*sRNAs used in this study.

Fig. S1. Cloning strategy workflow and final vector map

Fig. S2. Description of the 75-mer oligonucleotides for amiRNA and *Si*sRNA direct cloning in pUC18-AtMIR390a-B/c-RFP vector.

Fig. S3. Visualization of sRNA–mRNA predicted alignment.

Fig. S4. Expression of *S. indica*-specific genes in Arabidopsis roots inoculated with *S. indica,* as analysed by RT–PCR.

Fig. S5. Detection of *Si*sRNA21, *Si*sRNA24, and *Si*sRNA296 by multiplexed stem–loop PCR in Arabidopsis roots 7 d after inoculation with *S. indica*.

Fig. S6. Expression of amiRNAs or *Si*sRNAs in Arabidopsis leaf protoplasts.

Fig. S7. Relative accumulation of two *Si*sRNAs in transformed Arabidopsis protoplasts 24 hptr.

Fig. S8. 5ʹ-RLM-RACE for two Arabidopsis target genes of *Si*sRNA24 in Arabidopsis protoplasts.

Fig. S9. *At*AGO1/sRNA co-immunoprecipitation from *Si* axenic culture to show the specificity of the Arabidopsis AGO1 antibody.

Fig. S10. Comparison of target down-regulation by putative and artificial sRNA after their transient expression in Arabidopsis protoplasts.

Dataset S1. Information on *At* predicted targets of SisRNAs used in this study.

erae515_suppl_Supplementary_Tables_S1-S10_Figures_S1-S10

erae515_suppl_Supplementary_Dataset_S1

## Data Availability

All data associated with this paper are provided within the figures and supplementary data.

## Abbreviations:

AGO: ARGONAUTE
*At*: *Arabidopsis thaliana*
dpi: days post-inoculation
hptr: hours post-transformation
IP: immunoprecipitation
PTGS: post-transcriptional gene silencing
RISC: RNA-Induced Silencing Complex
*Si*: *Serendipita indica*
sRNA: small RNA
