# Supplementary material for: A pipeline for validation of Serendipita indica effector-like sRNA suggests cross-kingdom communication in the symbiosis with Arabidopsis
Source: J Exp Bot. 2024 Dec 26;76(6):1811–29. doi: 10.1093/jxb/erae515 (PMC11981902; doi:10.1093/jxb/erae515)
Supplement: erae515_suppl_Supplementary_Tables_S1-S10_Figures_S1-S10 [file erae515_suppl_supplementary_tables_s1-s10_figures_s1-s10.pdf]

10 Supplementary Tables and 10 Supplementary Figures

*Si*sRNA selection and Arabidopsis target prediction

In our previous work, Šečić *et al.* (2021) identified *Si*sRNAs in axenic fungal culture and *Si*sRNAs that were more abundant in colonized *B. distachyon* (*Bd*) roots using high-throughput sRNA sequencing. Among *Si*sRNAs with higher abundance in *Si* colonized *Bd* roots, 21 nt *Si*sRNAs were further analyzed, revealing 412 unique sequences from *Si* non-coding regions. For the current study, 14 *Si*sRNAs were selected based on their expression profile (expressed in axenic culture and during the interaction with *Bd*). Targets in *Arabidopsis thaliana* of these 14 *Si*sRNAs were *in-silico* predicted using psRNATarget (Scheme 2, 2017) (Dai *et al.,* 2018), , prioritizing those with interesting descriptions and where a single *Si*sRNA has multiple target genes. Supplementary Table S1 provides a summary of the 14 selected *Si*sRNAs, including extracted raw read counts, 5´-nucleotide, and their expression profile during the interaction with *Bd* extracted from Šečić et al., while Supplementary Dataset S2 Table S1 details their predicted targets in *At* as downloaded from *ps*RNATarget.

Supplementary Table S1. Information summary of the 14 *Si*sRNA selected for this study. Data extracted from Šečić *et al.* (2021) showing the 14 *Si*sRNA, their sequences, 5´-terminal nucleotide, raw read counts normalized to *Si*-colonized *Bd* roots or to *Si* axenic, and their expression profile during interaction of *Si* with *Bd*.

***Si*sRNA name**	***Si*sRNA sequence**	**5´-terminal nucleotide**	**Raw reads**	**Raw reads normalized to *Si-Bd* colonized roots**	**Raw reads normalized to *Si* axenic**	**Expression profile (*Bd* interaction)**
*Si*sRNA21	CAAATTTTGAATCTGGCGCCT	C	212	165.27	6.01	up
*Si*sRNA23	TACCCATACCTCGCCGTCGGC	T	95	74.06	4.42	up
*Si*sRNA24	TTGACCAATTTTTCTGTTCTC	T	57	44.43	4.56	up
*Si*sRNA25	TTGGAAGCGGCTGGACTAGTC	T	11	8.57	1.00	up
*Si*sRNA26	TACAACTTTCAACAACGGATC	T	82	63.92	14.73	up
*Si*sRNA28	ACAACTTTCAACAACGGATCT	A	595	463.85	109.6	up
*Si*sRNA37	GCTCACGTTCTATAGATTTGT	G	330	257.26	70.28	up

*Si*sRNA47	GGCCATGGAAGTCGGAACCCG	G	6	4.67	1.23	up
*Si*sRNA62	ATCCACGGCCATAGGACTCTG	A	2608	2033.18	856.15	up
*Si*sRNA86	CCGGGGTGTATTTATTAGATA	C	3	2.33	1.26	up
*Si*sRNA154	TCTACCTCTTGGTGAGCCGGC	T	35	27.28	0.47	up
*Si*sRNA165	TCGGGCAATGGCGCGAAGCTA	T	7	5.45	0.82	up
*Si*sRNA296	TCTGCGAATCGTAATTAAATA	T	2	1.55	0.26	up
*Si*sRNA376	TGATGCTCAGAACGGCGGCTC	T	2	1.55	#N/A	exclusive

Supplementary Table S2. Sequences of amiRNAs cloned in the pUC18-*At*MIR390a-B/c-RFP construct and expressed in *At* protoplasts for PTGS validation. Information on artificial microRNAs (amiRNAs) used in this study, their 5´- nucleotides, their corresponding predicted Arabidopsis target genes, their target predicted scores as indicated by psRNATarget (Dai *et al.,* 2018), and their mode of regulation. amiRNAs are designed using P.SAMS software (https://p-sams.carringtonlab.org) developed by the James Carrington lab (Carbonell *et al*. 2014). An expectation value indicates the penalty of mismatches between amiRNAs and the target sequence (*At* predicted target) with an expectation value of 0 being the highest level of sequence complementarity and 5 being the lowest.

**sRNA name**	**sRNA sequence 5´→ 3´**	**5´- terminal nucleotide**	**sRNA corresponding target**	**Target aligned fragment 5´→ 3´**	**Expectation value**	**Mode of regulation**
amir21	UACAUUCGCUC AGGUUCACCA	U	*AT5G25350*	AGGUGAACCUG AGCGAAUGUA	0	Cleavage
amir24	UCAAUCUCUAG AUGUUGACCA	U	*AT2G39020*	AGGUCAACAUC UAGAGAUUGA	0	Cleavage
amir154	UCCCUAGGAUA UUAUUGACAA	U	*AT4G32160*	AUGUCAAUAAU AUCCUAGGGA	0	Cleavage
amir296	UUGUUCAGGCG UUUUUAUCUG	U	*AT2G45240*	GAGAUAAAAAC GCCUGAACAA	0	Cleavage

Supplementary Table S3. Sequences of *Si*sRNA cloned in pUC18-*At*MIR390a-B/c-RFP construct and expressed into *At* protoplasts for PTGS validation. Information on *Si*sRNA used in this study, their 5´- nucleotides, their corresponding predicted Arabidopsis target genes, their expectation values indicating the degree of mismatches with the predicted

At target sequence as provided by psRNAtarget (Dai *et al.,* 2018) and their mode of regulation. An expectation value indicates the penalty of mismatches between mature small RNA (*Si*sRNA) and the target sequence (*At* predicted target), with a value of 0 being the highest level of sequence complementarity and 5 being the lowest.

sRNA name	sRNA sequence 5´→ 3´	5´-terminal nucleotide	predicted Arabidopsis target gene	Target aligned fragment 5´→ 3´	Expectation value	Mode of regulation
*Si*sRNA21	CAAAUUUUGAA UCUGGCGCCU	C	*AT1G01210*	AUGAGCCAGAAU CAAGAUUUU	4	Translation
			AT5G25350	AGGCGACGGAU UUGAGGUUGG	5	Cleavage
			AT5G37600	UCUUGCAAGAU UCAAAGUUUG	3	Cleavage
*Si*sRNA24	UUGACCAAUUU UUCUGUUCUC	U	*AT1G57590*	GAGGAAAGAGAA UUUGGUUAG	4.5	Cleavage
			AT1G63180	GAGAGUGGAAG AAUUGGAGAA	5	Cleavage
			AT1G65090	GAGUAUAGCAA GAUUGGUCAC	4.5	Cleavage
			AT4G15765	AAGAAUGGAAGA AUUGGACAU	4	Cleavage
			AT5G16680	GAGAACAGGAAG GAUGGUAAG	5	Cleavage
*Si*sRNA28	ACAACUUUCAAC AACGGAUCU	A	*AT1G05180*	AGAUUCAAUGU UGAAACUUGA	5	Cleavage
			AT3G06670	CGAUUUGGUGU UGGAGGUUGG	4	Cleavage
			AT5G55930	AGGUGUUGUUG UUGAAGGUUGU	4	Cleavage
*Si*sRNA154	UCUACCUCUUG GUGAGCCGGC	U	*AT2G47600*	GUUGACUUAUU AGGAGGUGGG	4.5	Cleavage
			AT4G32160	GUCUGCAUGCC AAGAUGUAGA	4.5	Cleavage

Supplementary Table S4: 75 mer oligonucleotides used for cloning *Si*sRNAs and amiRNAs into the pUC18-AtMIR390a-B/c-RFP vector.

Oligonucleotide name	75mer sequence
amir296-*AT2G45240*-75-F	TGTATTGTTCAGGCGTTTTTATCTGATGATGATCACATTCGTTAT CTATTTTTTCAGATAAAAAAGCCTGAACAA
amir296-*AT2G45240*-75-R	AATGTTGTTCAGGCTTTTTTATCTGAAAAAATAGATAACGAATGT GATCATCATCAGATAAAAACGCCTGAACAA

amir24-*AT2G39020*-75-F	TGTATCTTCACCGCTTGTTTGGCCTATGATGATCACATTCGTTATCTATTTTTTAGGCCAAACACGCGGTGAAGA
amir24-*AT2G39020*-75-R	AATGTCTTCACCGCGTGTTTGGCCTAAAAAATAGATAACGAATGTGATCATCATAGGCCAAACAAGCGGTGAAGA
amir21-*AT5G25350*-75-F	TGTATACATTCGCTCAGGTTCACCAATGATGATCACATTCGTTATCTATTTTTTTGGTGAACCTTAGCGAATGTA
amir21 -*AT5G25350* -75 -R	AATGTACATTCGCTAAGGTTCACCAAAAAAATAGATAACGAATGTGATCATCATTGGTGAACCTGAGCGAATGTA
amir154 -*AT4G32160* -75 -F	TGTATCCCTAGGATATTATTGACAAATGATGATCACATTCGTTATCTATTTTTTTTGTCAATAAGATCCTAGGGA
amir154 -*AT4G32160* -75 -R	AATGTCCCTAGGATCTTATTGACAAAAAAAAATAGATAACGAATGTGATCATCATTTGTCAATAATATCCTAGGGA
*Si*sRNA21 -75 -F	TGTACAAATTTTGAATCTGGCGCCTATGATGATCACATTCGTTATCTATTTTTTAGGCGCCAGAGTCAAAATTTG
*Si*sRNA21 -75 -R	AATGCAAATTTTGACTCTGGCGCCTAAAAAATAGATAACGAATGTGATCATCATAGGCGCCAGATTCAAAATTTG
*Si*sRNA24 -75 -F	TGTATTGACCAATTTTTCTGTTCTCATGATGATCACATTCGTTATCTATTTTTTGAGAACAGAACAATTGGTCAA
*Si*sRNA24 -75 -R	AATGTTGACCAATTGTTCTGTTCTCAAAAAATAGATAACGAATGTGATCATCATGAGAACAGAAAAATTGGTCAA
*Si*sRNA28 -75 -F	TGTAACAACTTTCAACAACGGATCTATGATGATCACATTCGTTATCTATTTTTTAGATCCGTTGGTGAAAGTTGT
*Si*sRNA28 -75 -R	AATGACAACTTTCACCAACGGATCTAAAAAATAGATAACGAATGTGATCATCATAGATCCGTTGTTGAAAGTTGT
*Si*sRNA154 -75 -F	TGTATCTACCTCTTGGTGAGCCGGCATGATGATCACATTCGTTATCTATTTTTTGCCGGCTCACAAAGAGGTAGA
*Si*sRNA154 -75 -R	AATGTCTACCTCTTTGTGAGCCGGCAAAAAATAGATAACGAATGTGATCATCATGCCGGCTCACCAAGAGGTAGA

Supplementary Table S5: Primers used in stem-loop End-point PCR and stem-loop qPCR (cDNAhp = complementary DNA hairpin, F = forward)

**Primer name**	**Primer sequence**
amir296-*AT2G45240*-cDNAhp	GTCGTATCCAGTGCAGGGTCCGAGGTATTCGCACTGGATACGACcagata
amir296-*AT2G45240*-F	TCGCTttgttcaggcgtttt
amir24-*AT2G39020*-cDNAhp	GTCGTATCCAGTGCAGGGTCCGAGGTATTCGCACTGGATACGACaggcca
amir24-*AT2G39020*-F	TCGCTtcttcaccgcttgtt
amir21-*AT5G25350*-cDNAhp	GTCGTATCCAGTGCAGGGTCCGAGGTATTCGCACTGGATACGACtggtga
amir21-*AT5G25350*-F	TCGCTtacattcgctcaggt
amir154-*AT4G32160*-cDNAhp	GTCGTATCCAGTGCAGGGTCCGAGGTATTCGCACTGGATACGACttgtca
amir154-*AT4G32160*-F	TCGCTtccctaggatattat
*Si*sRNA21-cDNAhp	GTCGTATCCAGTGCAGGGTCCGAGGTATTCGCACTGGATACGACaggcgc
*Si*sRNA21-F	TCGCTcaaattttgaatctg

*Si*sRNA24-cDNAhp	GTCGTATCCAGTGCAGGGTCCGAGGTATTCGCACTGGATACGACg agaac
*Si*sRNA24-F	TCGCTttgaccaatttttct
*Si*sRNA28-cDNAhp	GTCGTATCCAGTGCAGGGTCCGAGGTATTCGCACTGGATACGACa gatcc
*Si*sRNA28-F	TCGCTacaactttcaacaac
*Si*sRNA-154-cDNAhp	GTCGTATCCAGTGCAGGGTCCGAGGTATTCGCACTGGATACGACg ccggc
*Si*sRNA154-F	TCGCTtctacctcttggtga
*At*miR159a-cDNAhp	GTCGTATCCAGTGCAGGGTCCGAGGTATTCGCACTGGATACGACt agagc
*At*miR159a-F	TCGCTtttggattgaaggga
*At*miR166a-cDNAhp	GTCGTATCCAGTGCAGGGTCCGAGGTATTCGCACTGGATACGACg gggaa
*At*miR166a-F	TCGCTtcggaccaggcttca
Univ-stemloop-reverse	GTATCCAGTGCAGGGTCCGAGGT

Supplementary Table S6: Primers used for gene expression analysis in quantitative real-time PCR (qPCR).

Primer name	Primer sequence
q*AT1G01210* -F	CTGAAACTGAAGCCCCATGT
q*AT1G01210* -R	ATTCCTCACGCCAAGTGAAC
q*AT5G25350* -F	AGGAGTTTGGAGGGGAAGAA
q*AT5G25350* -R	ATGGACAACCATGAGCAACA
q*AT5G37600* -F	CAATCCTCTGGAATCCTTGA
q*AT5G37600* -R	AAAAGCAGAATAAGCAGAGCAAA
q*AT1G57590* -F	TTGTGACGGTGGATCGTTTA
q*AT1G57590* -R	AGCAGAGCCTGCTTAGCTTG
q*AT1G63180* -F	GCGGAACCAGAATGGAAGAT
q*AT1G63180* -R	TTATTCGGTATGCCCTTTGG
q*AT1G65090* -F	AGTTGGCAAGGAGGATGATG
q*AT1G65090* -R	TGAATGGGAATGGGTTCTTC
q*AT4G15765* -F	CGACATTGCAGGACATAACG
q*AT4G15765* -R	CCTGTGAAGAGCAAGGGAAG
q*AT5G16680* -F	TTTGCAGAATCCATCATCCA
q*AT5G16680* -R	TCCTGAACGCGTAGATCCTT
q*AT2G39020* -F	TTTGTCCAAGCCTGGTTTTT
q*AT2G39020* -R	TTCCAATCAAGAACAACCCATT
qA*T1G05180* -F	AGTGTTGGCCAATCAAAAGC
q*AT1G05180* -R	GCCATAAGAGCGAACCAAAA
q*AT3G06670* -F	CTCGTTGCTCCAAAACCCTA
q*AT3G06670* -R	GGAGCGCCCATAACTGATTA
q*AT5G55930* -F	GGGCTTGAGAATTCAGACCA
q*AT5G55930* -R	TTACCCCTTTCAGGACAACG
q*AT2G47600* -F	CTCCGATTCTTTTCCCCAGT
q*AT2G47600* -R	TTCTTGCAACTCCTGGGTTT
q*AT4G32160* -F	GGTGCTCTCTTGAGGAGTGG

q*AT4G32160* -R	ATAGGCGACGAACTTGTGCT
q*AT2G45240* -F	AAGGCCAGGCTAGAACACCT
q*AT2G45240* -R	TCACGCATTCTCTGGATTTG
qUBC21 -F	GCTTGGAGTCCTGCTTGGACG
qUBC21-R	CGCAGTTAAGAGGACTGTCCGGC
*At*EF1α-F	CTGTTGTAACAAGATGGATGCC
*At*EF1α-R	CCCTCGAATCCAGAGATTGG

Supplementary Table S7: Primers used for RNA ligase-mediated rapid amplification of cDNA ends (RLM-RACE)

Primer Name	Primer sequence
RLM -*AT2G45240* -Outer -Specific	ATCAGGCCATGTTCGATCCC
RLM -*AT2G45240* -Inner -Specific	TTGTAGCATGGCGGTTGAC
RLM -*AT1G15765* -Outer -Specific	TTAACGTGCACAATCGGAAA
RLM -*AT1G15765* -Inner -Specific	GCCAAGAGGAAGAGCATGAG
RLM -*AT1G65090* -Outer -Specific	CCTCATCGTCCTTGCCCGTAGAA
RLM -*AT1G65090* -Inner -Specific	CTTTACCCCTGAAAAGTGCAGCC
RLM -Outer -Universal	GCTGATGGCGATGAATGAACACTG
RLM -Inner -Universal	GAACACTGCGTTTGCTGGCTTTGATG

Supplementary Table S8: PCR primers

Primer Name	Primer sequence
Si-ITS -Fwd	CAACACATGTGCACGTCGAT
Si-ITS -Rev	CCAATGTGCATTCAGAACGA
Si -Ubi -Fwd	GCAGCTCGAAGATGGTCGC
Si -Ubi -Rev	ACATGCACGCTTGCGGAGT
M13 -Fwd	GTTTTCCCAGTCACGAC
M13 -Rev	AACAGCTATGACCATG
Attb -Fwd	GGGGACAAGTTTGTACAAAAAAGCAGGCT
Attb -Rev	GGGGACCACTTTGTACAAGAAAGCTGGGT
pUC18-Mut-Fwd	GCAATGATACCGCGAGAGCCACGCTCACCGGCTCC
pUC18-Mut-Rev	GGAGCCGGTGAGCGTGGCTCTCGCGGTATCATTGC

Red nucleotides present the substituted bases for site-directed mutagenesis incorporated in the restriction site of BsaI in the backbone of the pUC18 vector (see material and methods).

Supplementary Table S9. Sequences of *Si*sRNA detected in *Si* axenic and *Si-At* interaction compared to the respective stem-loop PCR amplicons detected from *Si-At* Co-IP.

sRNA name	*Si*sRNA sequence detected in *Si* axenic and/or *Si-At*	Sequence of *Si*sRNA detected in Co-IP
*Si*sRNA21	CAAATTTTGAATCTGG CGCCT	TCGCT**CAAATTTTGAATCTGTCGATC**CAGTGCGAATACCTC GGACCCTGCACTGGATACAATCGAATTCCCGCGGCCGCC
*Si*sRNA23	TACCCATACCTCGCCG TCGGC	TCGCT**TACCCATACCTCGCCGTCGTA**TCCAGTGCGAATACCT CGGACCCTGCACTGGATACAATCACTAGTGAATTCGCG
*Si*sRNA24	TTGACCAATTTTTCTGT TCTC	TCGCT**TTGACCAATTTTTCTGTTCTC**GTCCGTATCCAGTGCG AATACCTCGGACCCTGCACTGGATACAATCGAATTCCC
*Si*sRNA28	ACAACTTTCAACAACG GATCT	TCGCT**ACAACTTTCAACAACGGATCT**GTCGTATCCAGTGCG AATACCTCGGACCCTGCACTGGATACAATCACTAGTGAA
*At*miR159a	TTTGGATTGAAGGGA GCTCTA	GCGCC**TTTGGATTGAAGGGAGCTCTA**GTCGTATCCAGTGC GAATACCTCGGACCCTGCACTGGATACAATCGAATTCCCG

Bold letters highlight the presence of the 21 nt *Si*sRNA sequences (detected in *Si* axenic and *Si-At*) in the stem-loop PCR amplicon detected from Co-IP. **Red letters** indicate different nucleotides of *Si*sRNAs from the Co-IP sequencing results relative to the sequencing results of *Si*sRNA in the axenic culture.

Supplementary Table S10. Summary of the selected *Si*sRNAs used in this study. The table provides a summary of *Si*sRNAs analysed, including their predicted and experimentally confirmed targets, functional roles, and the assay used for the validation of the *Si*sRNA and their corresponding targets. Targets were *in silico* predicted using psRNATarget, while confirmed downregulated targets were validated via qPCR in transformed protoplasts, *Si-Ath* interaction, or both. Targets highlighted in yellow indicate downregulation in *Si-Ath* interaction at 7 dpi, green indicated downregulation in transformed protoplasts, and blue indicates downregulated in both.

*Si*sRNA name	sRNA detection assay	Selected predicted-investigated targets	Functional role	sRNA-target interaction assay
*Si*sRNA21	- sRNA seq (Šečić et al, 2021)<br>- SL-qPCR	(3 out of 43)<br>- *AT1G01210*<br>- *AT5G25350*<br>- *AT5G37600*	- EIN3-binding F box protein 2 (ethylene response pathway)<br>- ARABIDOPSIS GLUTAMINE SYNTHASE (Regulate nitrogen assimilation)	- qPCR
*Si*sRNA24	- sRNA seq (Šečić et al, 2021)<br>- SL-qPCR<br>-AGO1/sRNA Co-IP	(5 out of 82)<br>- *AT1G57590*<br>- *AT1G63180*<br>- *AT1G65090*<br>- *AT4G15765*<br>- *AT5G16680*	- Pectinacetylesterase family protein (cell wall organization)<br>- UDP-D-GLUCOSE (pollen development)<br>- SEED LIPID DROPLET PROTEIN1 (protein binding)<br>- FAD/NAD(P)-binding oxidoreductase<br>- RING/FYVE/PHD zinc finger superfamily protein (regulation of gene expression)	- qPCR
*Si*sRNA28	- sRNA seq (Šečić et al, 2021)<br>- SL-qPCR<br>-AGO1/sRNA Co-IP	(3 out 49)<br>- *AT1G05180*<br>- *AT3G06670*<br>- *AT5G55930*	- AUXIN RESISTANT 1 (significant role in DNA repair)<br>- PLATINUM SENSITIVE 2 LIKE (regulatory ncRNA processing)<br>- ARABIDOPSIS THALIANA OLIGOPEPTIDE	- qPCR

			TRANSPORTER 1 (oligopeptide transmembrane transporter activity)	
*Si*sRNA154	- sRNA seq (Šečić et al, 2021)<br>- SL-qPCR	(2 out of 25)<br>- *AT2G47600*<br>- *AT4G32160*	- MAGNESIUM/PROTON EXCHANGER (iron, magnesium and zinc ion transport)<br>- Phox (PX) domain-containing protein (signal transduction)	- qPCR

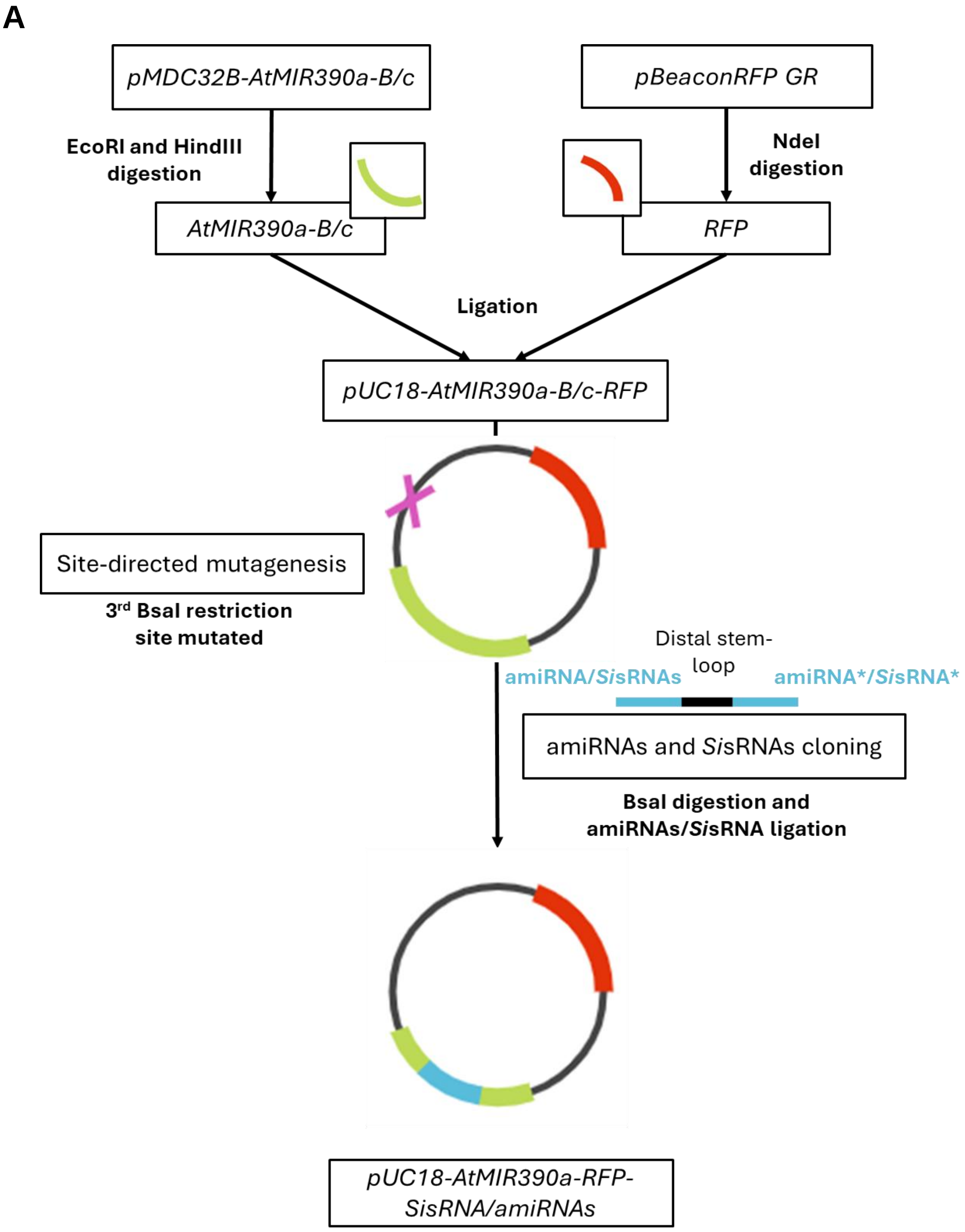
A
pMDC32B-AtMIR390a-B/c
pBeaconRFP GR
EcoRI and HindIII digestion
NdeI digestion
AtMIR390a-B/c
RFP
Ligation
pUC18-AtMIR390a-B/c-RFP
Site-directed mutagenesis
3rd BsaI restriction site mutated
Distal stem-loop
amiRNA/SisRNAs
amiRNA*/SisRNA*
amiRNAs and SisRNAs cloning
BsaI digestion and amiRNAs/SisRNA ligation
pUC18-AtMIR390a-RFP-SisRNA/amiRNAs

B

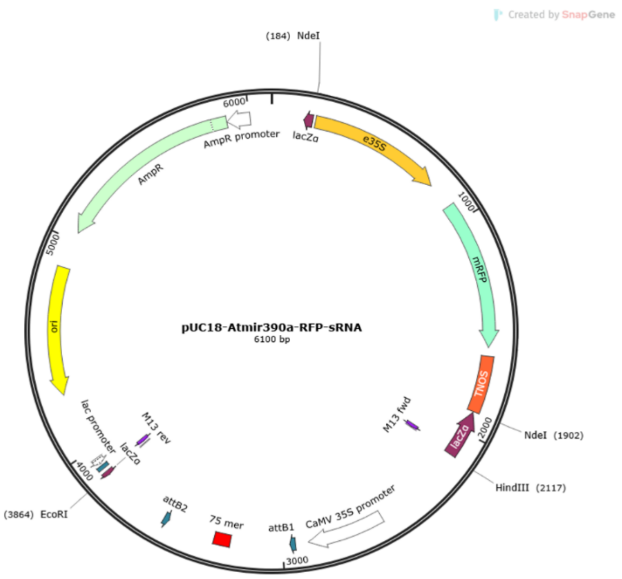

Supplementary Fig. S1. Cloning strategy workflow and final vector map. **(A)** Illustration of the cloning strategy workflow. **(B)** Final pUC18-*At*MIR390a-RFP-amiRNA/*Si*sRNA vector map. The *At*MIR390a-B/c fragment was subcloned from the pMDC32B-*At*MIR390a-B/c vector (Plasmid #51776, Carbonell et al., 2014) into pUC18 (Plasmid #50004), using *EcoRI* and *HindIII*, resulting in pUC18 -*At*MIR390a-B/c. The *CaMV35S::RFP* expression cassette was extracted from the pBeaconRFP GR vector (https://gatewayvectors.vib.be/index.php/ ID: 3_20, Bargmann and Birnbaum (2009) and cloned into pUC18-*At*MIR390a-B/c using *Nde*I, resulting in pUC18-*At*MIR390a-B/c-RFP. A *Bsa*I restriction site in the backbone of pUC18-*At*MIR390a-B/c-RFP was removed by site-directed mutagenesis. 75mer oligos encoding 21nt sRNA (amiRNAs or *Si*sRNAs) sequences were annealed and cloned into the *Bsa*I sites of pUC18-*At*MIR390a-B/c-RFP (flanking the *At*MIR390a-B/c insert) by GoldenGate cloning, resulting in the final pUC18-*At*MIR390a-RFP-sRNA expression vector. *In-silico* cloning was created with SnapGene software. Vector maps are available at https://www.addgene.org/.

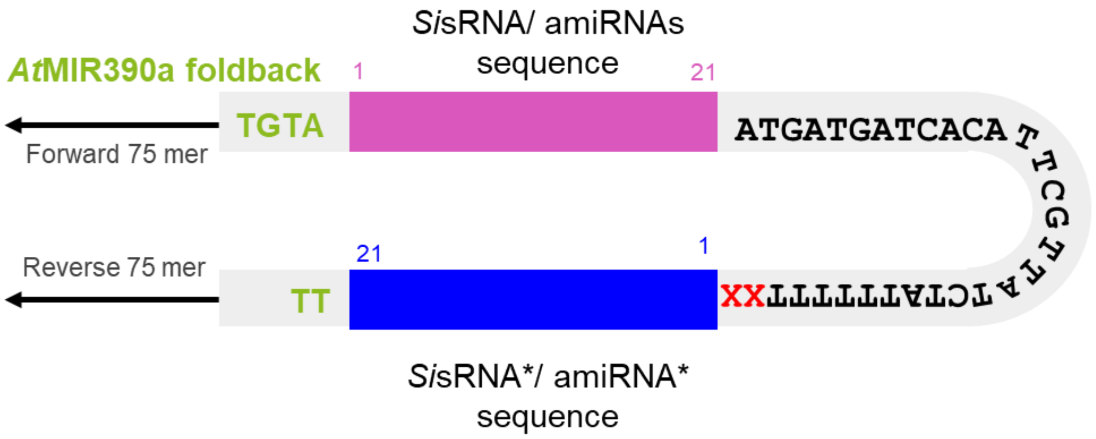

Supplementary Fig. S2: Description of the 75-mer oligonucleotides content (adapted from Carbonell *et al.*, 2014) for amiRNAs and *Si*sRNA direct cloning in pUC18-AtMIR390a-B/c-RFP vector. 75-mer Forward and reverse overlapping oligonucleotides contain the *Si*sRNA/amiRNA sequence (in pink) and the *Si*sRNA*/amiRNA* sequence (in blue), respectively. Nucleotides in green are the base of *At*MIR390a foldback included in the 75-mer oligonucleotides required for the cloning into the vector, and in red are the nucleotides that may be modified to preserve the authentic *At*MIR390a duplex structure.

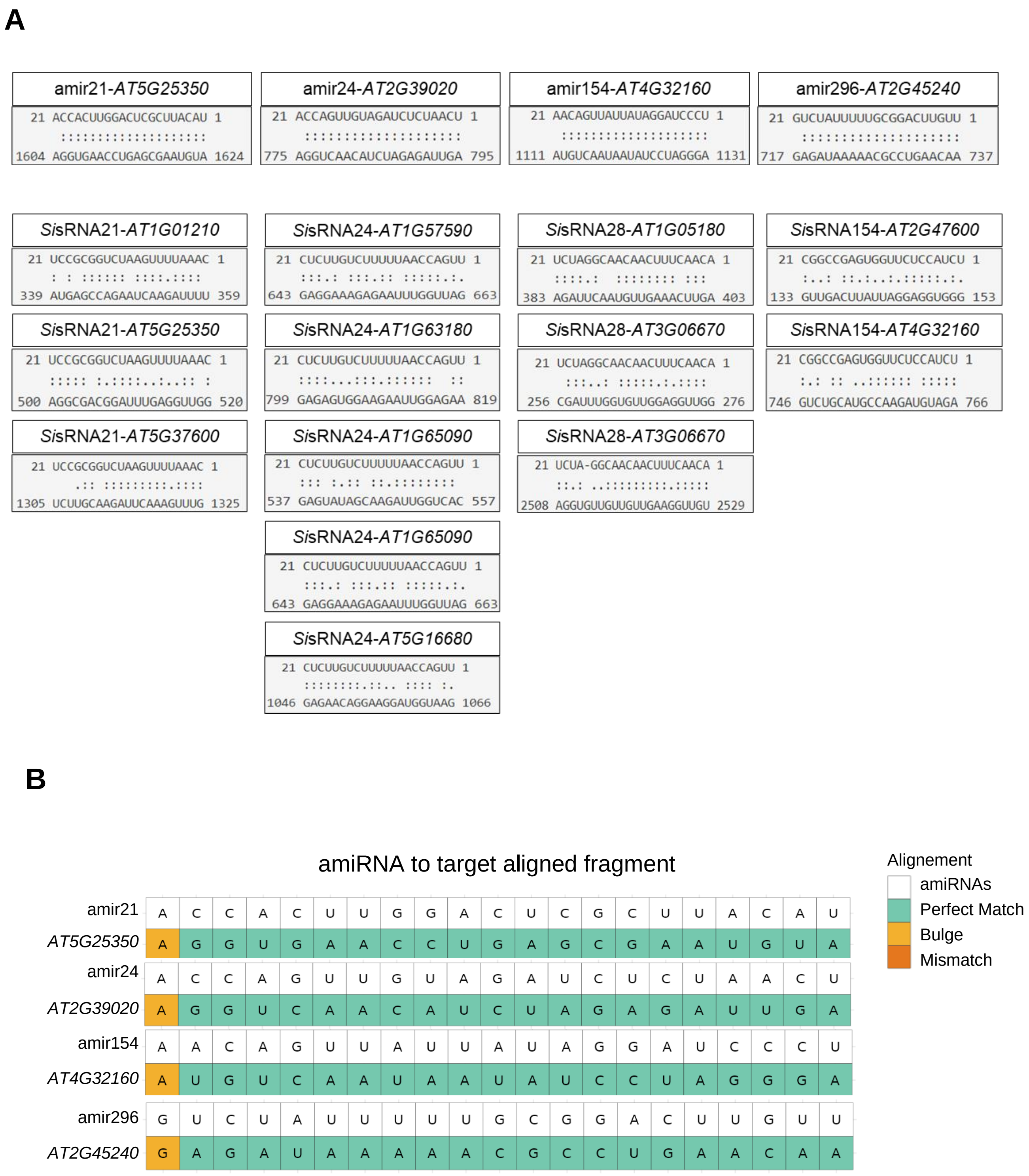
A
amir21-AT5G25350
21 ACCACUUGGACUCGCUUACAU 1
1604 AGGUGAACCUGAGCGAAUGUA 1624
amir24-AT2G39020
21 ACCAGUUGUAGAUCUCUAACU 1
775 AGGUCAACAUCUAGAGAUUGA 795
amir154-AT4G32160
21 AACAGUUAUUAUAGGAUCCCU 1
1111 AUGUCAAUAAUAUCCUAGGGA 1131
amir296-AT2G45240
21 GUCUAUUUUUGCGGACUUGUU 1
717 GAGAUAAAAACGCCUGAACAA 737
SisRNA21-AT1G01210
21 UCCGCGGUCUAAGUUUUAAAC 1
339 AUGAGCCAGAAUCAAGAUUUU 359
SisRNA24-AT1G57590
21 CUCUUGUCUUUUUAACCAGUU 1
643 GAGGAAAGAGAAUUUGGUUAG 663
SisRNA28-AT1G05180
21 UCUAGGCAACAACUUUCAACA 1
383 AGAUUCAAUGUUGAAACUUGA 403
SisRNA154-AT2G47600
21 CGGCCGAGUGGUUCUCCAUCU 1
133 GUUGACUUAUUAGGAGGUGGG 153
SisRNA21-AT5G25350
21 UCCGCGGUCUAAGUUUUAAAC 1
500 AGGCGACGGAUUUGAGGUUGG 520
SisRNA24-AT1G63180
21 CUCUUGUCUUUUUAACCAGUU 1
799 GAGAGUGGAAGAAUUGGAGAA 819
SisRNA28-AT3G06670
21 UCUAGGCAACAACUUUCAACA 1
256 CGAUUUGGUGUUGGAGGUUGG 276
SisRNA154-AT4G32160
21 CGGCCGAGUGGUUCUCCAUCU 1
746 GUCUGCAUGCCAAGAUGUAGA 766
SisRNA21-AT5G37600
21 UCCGCGGUCUAAGUUUUAAAC 1
1305 UCUUGCAAGAUUCAAAGUUUG 1325
SisRNA24-AT1G65090
21 CUCUUGUCUUUUUAACCAGUU 1
537 GAGUAUAGCAAGAUUGGUCAC 557
SisRNA28-AT3G06670
21 UCUA-GGCAACAACUUUCAACA 1
2508 AGGUGUGUUGUUGAAGGUUGU 2529
SisRNA24-AT1G65090
21 CUCUUGUCUUUUUAACCAGUU 1
643 GAGGAAAGAGAAUUUGGUUAG 663
SisRNA24-AT5G16680
21 CUCUUGUCUUUUUAACCAGUU 1
1046 GAGAACAGGAAGGAUGGUAAG 1066
B
amiRNA to target aligned fragment
amir21 A C C A C U U G G A C U C G C U U A C A U
AT5G25350 A G G U G A A C C U G A G C G A A U G U A
amir24 A C C A G U U G U A G A U C U C U A A C U
AT2G39020 A G G U C A A C A U C U A G A G A U U G A
amir154 A A C A G U U A U U A U A G G A U C C C U
AT4G32160 A U G U C A A U A A U A U C C U A G G G A
amir296 G U C U A U U U U U G C G G A C U U G U U
AT2G45240 G A G A U A A A A A C G C C U G A A C A A
Alignement
amiRNAs
Perfect Match
Bulge
Mismatch

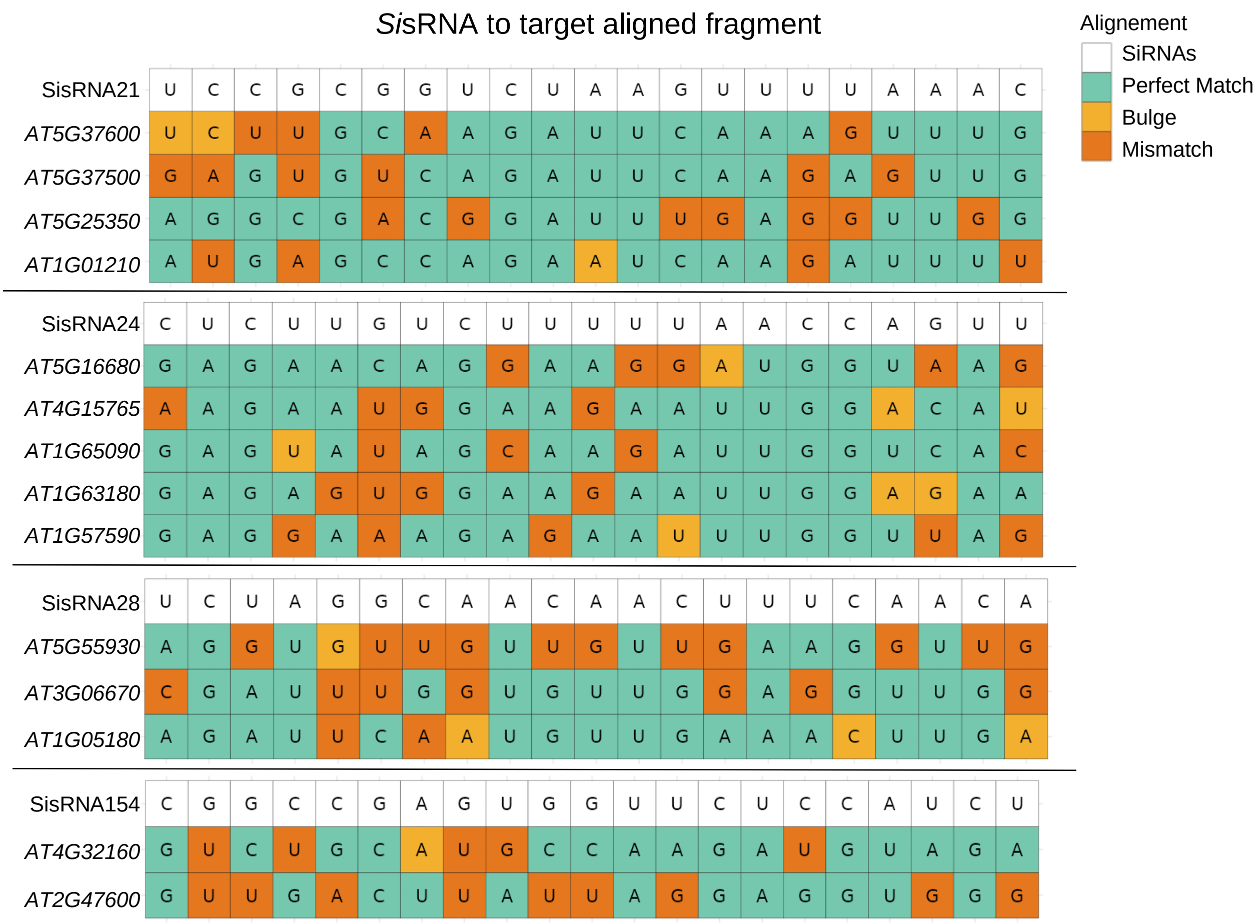

Supplementary Fig. S3. Visualisation of sRNA/mRNA predicted alignement. **(A)** Illustration of the alignment between the investigated amiRNAs and *Si*sRNA and their predicted Arabidopsis target genes as displayed by psRNATarget web tool. Each panel displays a sequence alignment, with the query sequence (21 nt amiRNA or *Si*sRNA) shown above and the target predicted fragment shown below. Perfect matches are represented by (:), while (.) or ( ) indicates a bulge or a mismatch. **(B)** A color coded heatmap alignment displaying the nucleotide complementarity between amiRNAs or *Si*sRNA and their predicted targets used in this study. The alignment heatmap was generated using ggplot library in R (version 2023, 4.3.2).

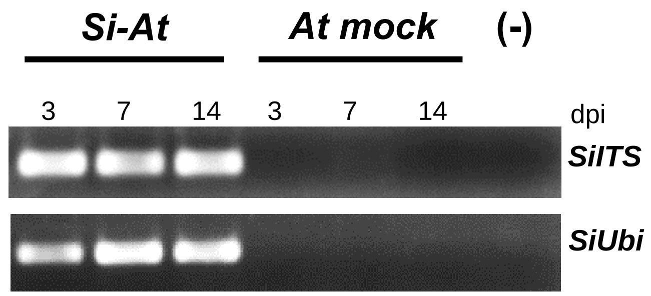

Supplementary Fig. S4. Expression of *S. indica*-specific genes in Arabidopsis roots inoculated with *Si,* as analysed by RT-PCR. The expression of the *Internal Transcribed Spacer* (*SiITS*) and of *Si Ubiquitin (SiUbi)* genes was examined at 3-, 7- and 14 dpi of Arabidopsis roots inoculated with *Si* chlamydospores. Arabidopsis roots treated with Tween 20 (0.002% in H_2O) were used as a control (mock). (-) indicate a non-template control.

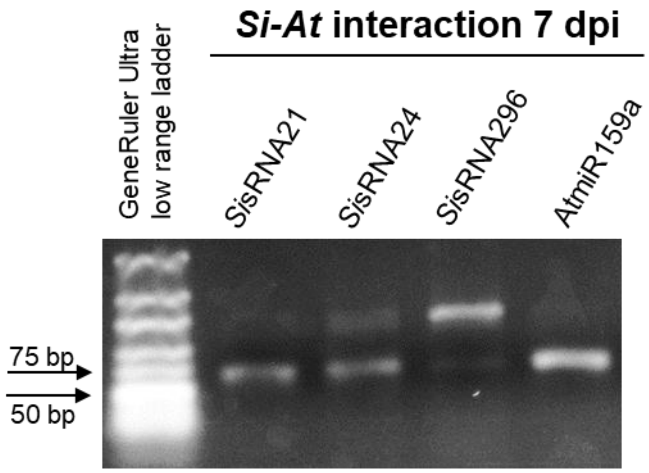

Supplementary Fig. S5. Detection of *Si*sRNA2, *Si*sRNA24 and *Si*sRNA296 by multiplexed stem-loop PCR (cDNA generated from multiple hairpin sRNA primers simultaneously in one reaction) in Arabidopsis roots 7 dpi with *S. indica*. A PCR product of the expected size of 62 bp (comprising the 21 bp of the sRNA sequence, loop region, plus the forward primer and the reverse primer length) is visualized in a 2% agarose gel. The plant 21 nt *At*miR159a was used as an endogenous control.

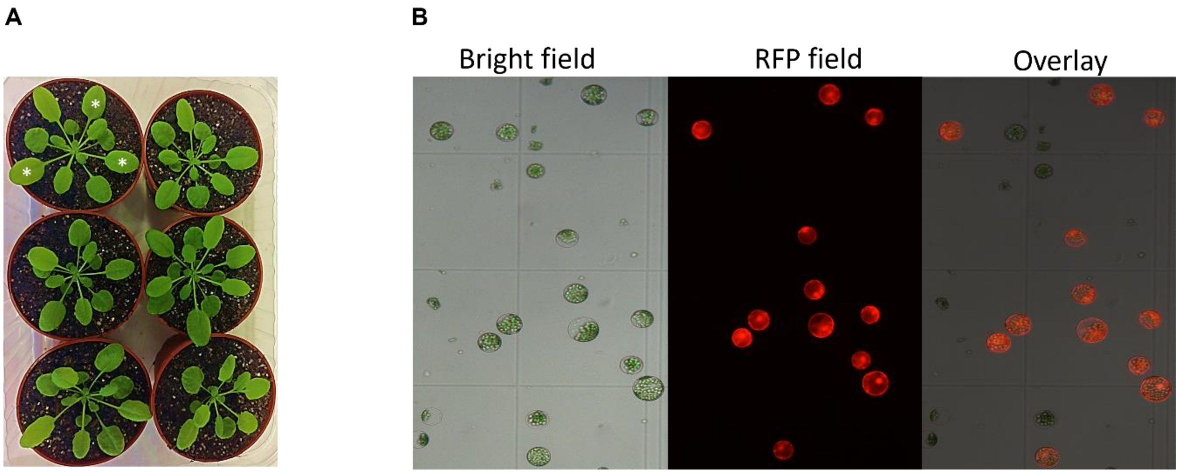

Supplementary Fig. S6. Expression of amiRNAs or *Si*sRNAs in Arabidopsis leaf protoplasts. **(A)** 4- to 5-week-old Arabidopsis (Col-0) plants suitable for protoplast isolation. (*) optimal leaves to be used for protoplast isolation. **(B)** Arabidopsis leaf protoplasts one day after transformation with the expression vector pUC18-*At*MIR390-RFP-sRNA, containing a red fluorescing protein (RFP) gene under the control of the CaMV35S promoter, to confirm the successful transformation. Red protoplasts in the RFP image carry the expression vector.

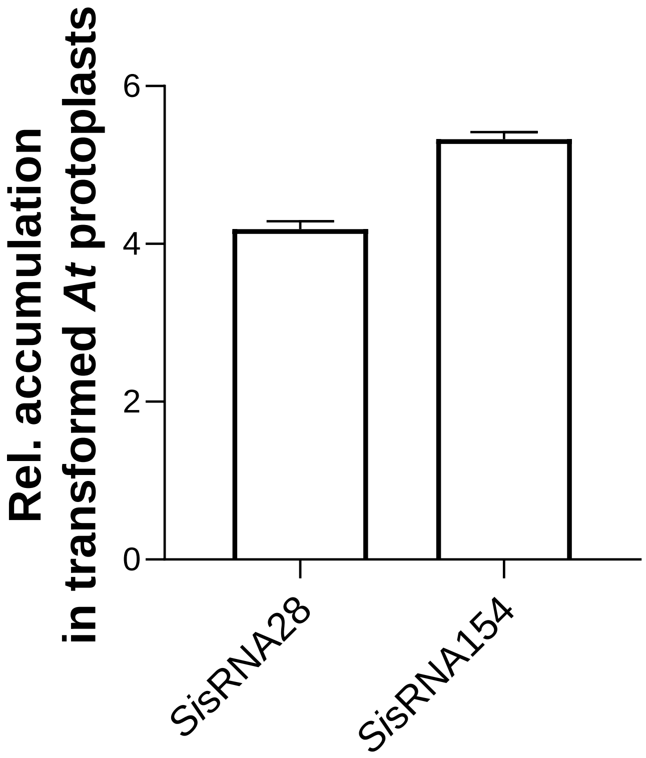

Supplementary Fig. S7. Relative accumulation of two *Si*sRNAs in transformed Arabidopsis protoplasts 24 hptr. The relative amount of *Si*sRNA28 and *Si*sRNA154 were normalized to *At*miR159a. Values represent the mean ± SE of three technical replicates from one biological replicate.

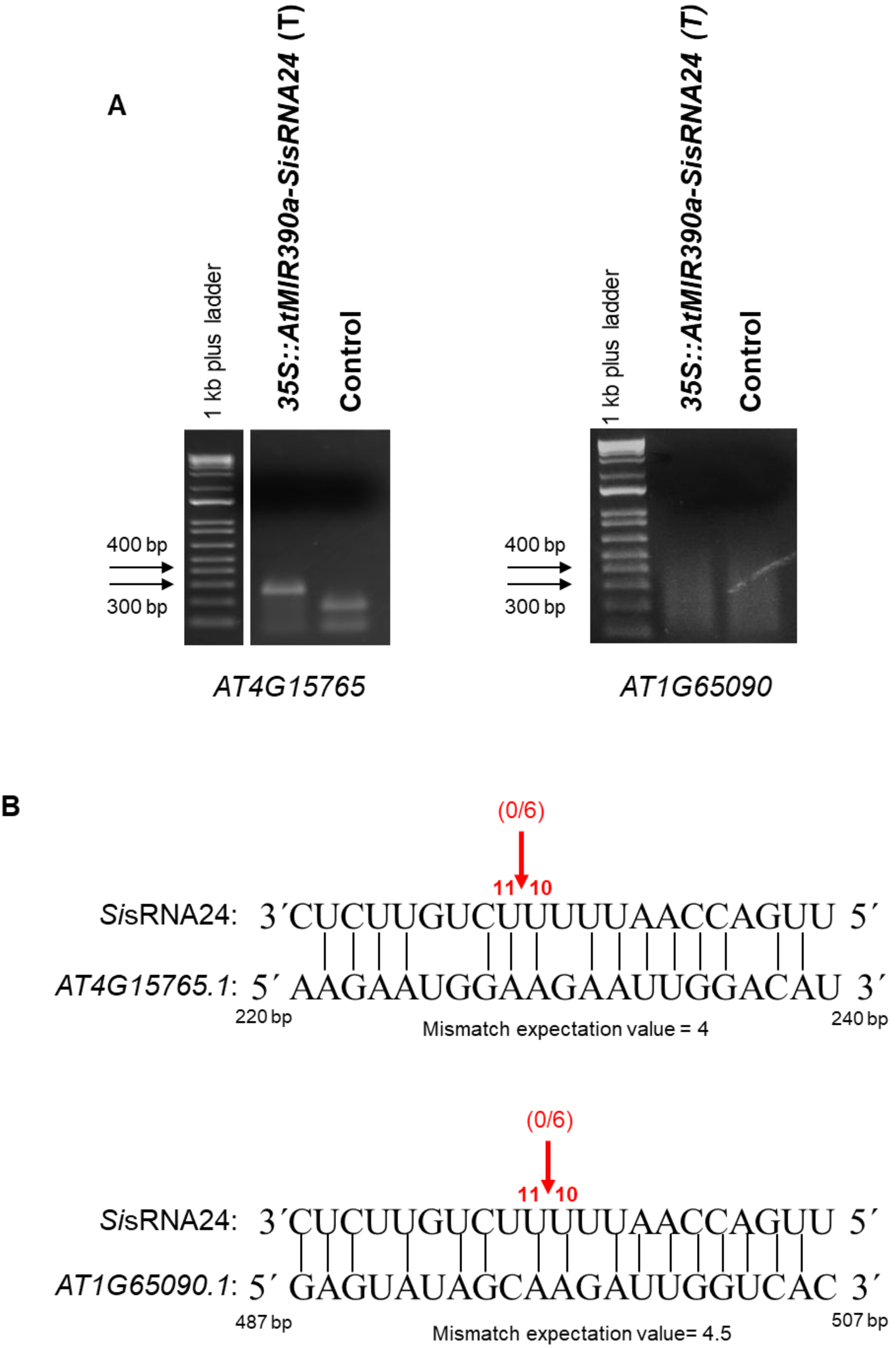
A
1 kb plus ladder
35S::AtMIR390a-SisRNA24 (T)
Control
400 bp
300 bp
AT4G15765
1 kb plus ladder
35S::AtMIR390a-SisRNA24 (T)
Control
400 bp
300 bp
AT1G65090
B
(0/6)
11 10
SisRNA24: 3´CUCUUGUCUUUUUAACCAGUU 5´
AT4G15765.1: 5´ AAGAAUGGAAGAAUUGGACAU 3´
220 bp
240 bp
Mismatch expectation value = 4
(0/6)
11 10
SisRNA24: 3´CUCUUGUCUUUUUAACCAGUU 5´
AT1G65090.1: 5´ GAGUAUAGCAAGAUUGGUCAC 3´
487 bp
507 bp
Mismatch expectation value= 4.5

Supplementary Fig. S8. 5' RLM-RACE for two Arabidopsis target genes of *Si*sRNA24 in Arabidopsis protoplasts. RNA was extracted from Arabidopsis protoplasts 24 hptr with an expression construct of *Si*sRNA24. Water replaced *Si*sRNA24 in protoplasts served as control. **(A)** PCR fragments after 5' RLM-RACE for the two *Si*sRNA24 targets *AT4G15765* and *AT1G65090* as visualised by Agarose gel electrophoresis. No clear PCR amplicon was detected for *AT1G65090* **(B)** Predicted mapping of *AT4G15765* and *AT1G65090* cleavage sites. Red arrows indicate the expected canonical cleavage sites. The proportion of cloned 5´-RLM-RACE products at the predicted cleavage sites is shown in brackets. The expectation value (also called the target predicted score, TPS) as predicted by the psRNATarget server (http://plantgrn.noble.org/psRNATarget/) indicates the penalty for the mismatches between mature sRNA (*Si*sRNA) and the target sequence (*At* predicted target) with TPS of 0 is the highest level of sequence complementarity, and 5 is the lowest.

A

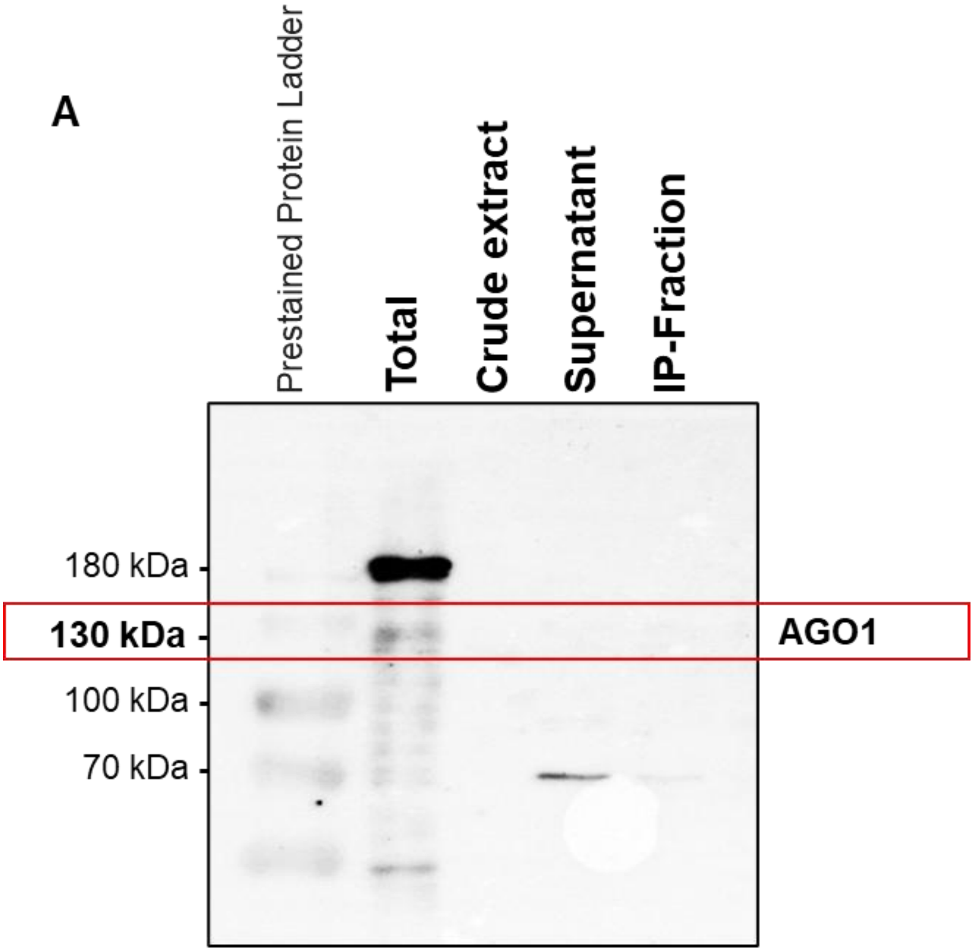
Prestained Protein Ladder
Total
Crude extract
Supernatant
IP-Fraction
180 kDa
130 kDa
100 kDa
70 kDa
AGO1

B

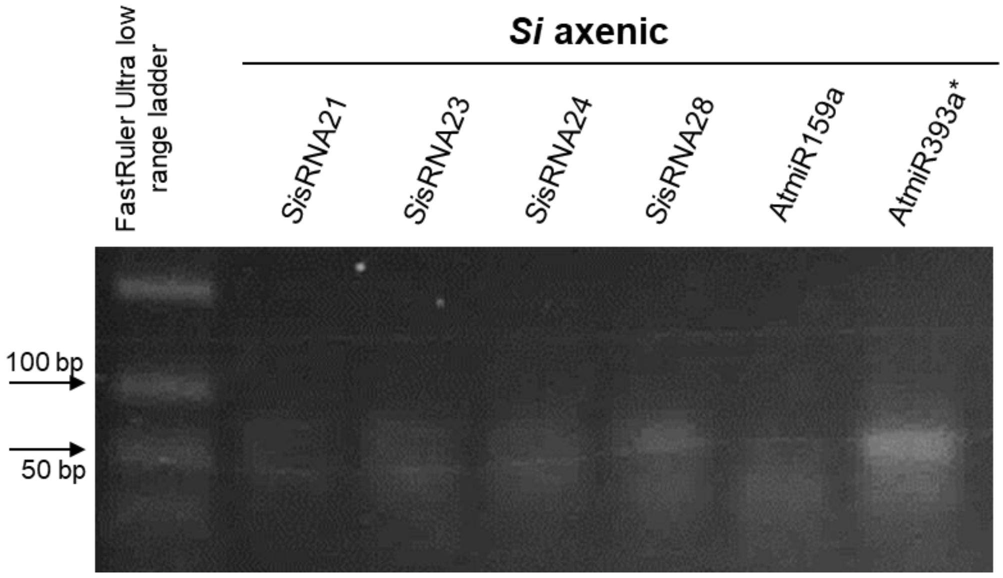
FastRuler Ultra low range ladder
Si axenic
SisRNA21
SisRNA23
SisRNA24
SisRNA28
AtmiR159a
AtmiR393a*
100 bp
50 bp

Supplementary Fig. S9. *At*AGO1/sRNA co-immunoprecipitation from *Si* axenic culture to show the specificity of the Arabidopsis AGO1 antibody. **(A)** Total protein was isolated from *Si* and grown in an axenic culture for 4 weeks, and immunoprecipitation was performed using an anti-*At*AGO1 antibody. No signal could be detected in the *Si* samples, excluding cross-reaction of the anti-*At*AGO1 antibody with *Si* AGO proteins. Weak unspecific bands of 70 kDa were detected in the SN and the IP fraction suggesting an unspecific binding to unrelated proteins or the presence of a contaminant. **(B)** Stem-loop PCR to detect *Si*sRNAs recovered from *At*AGO1/sRNA co-immunoprecipitation from *Si* axenic culture. No amplification or weak bands of primer dimers were obtained. Arabidopsis *At*miR159a and *At*miR393a* were used as negative controls.

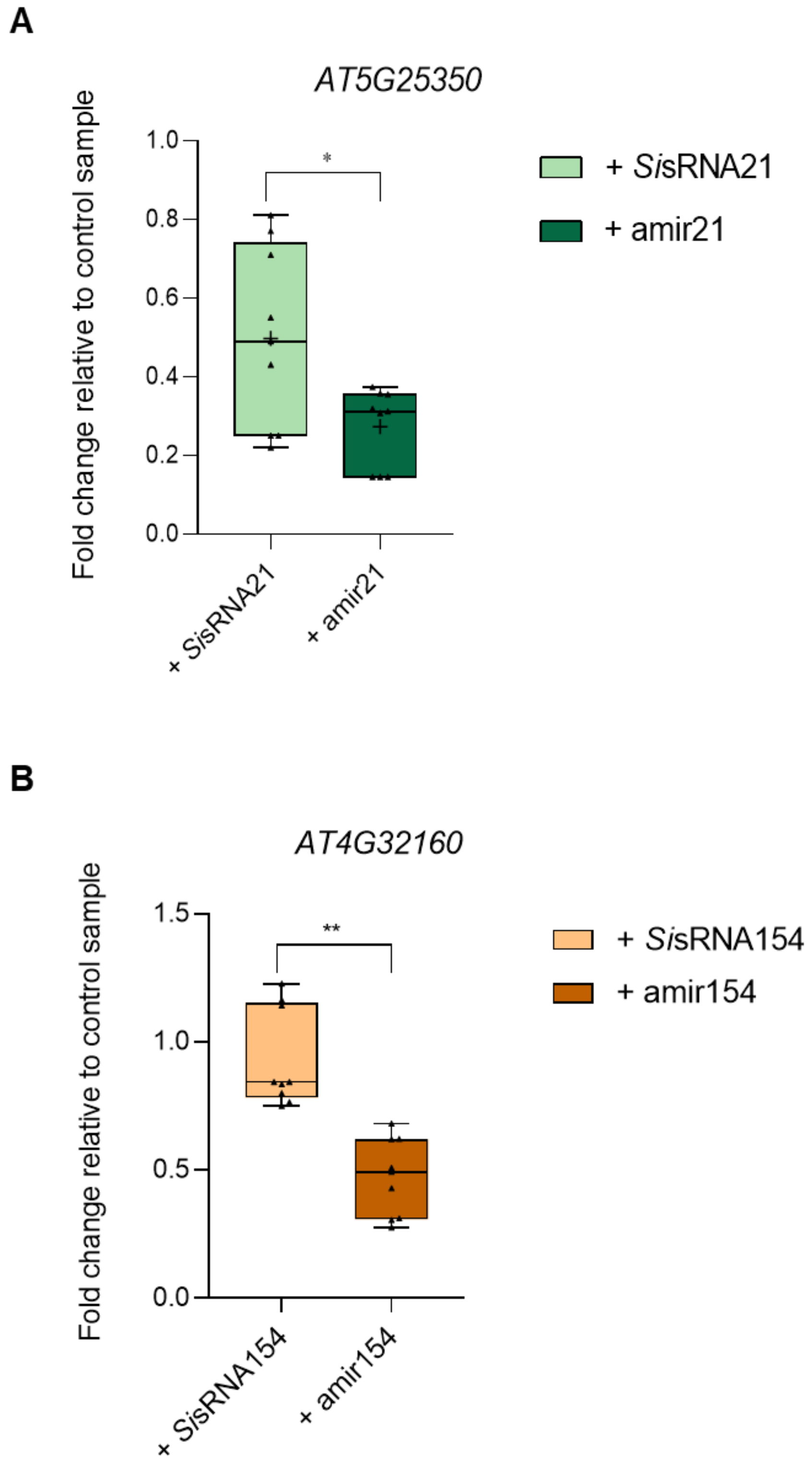

Supplementary Fig. S10. Comparison of target downregulation by putative and artificial sRNA after their transient expression in Arabidopsis protoplasts. **(A)** Comparison of *Si*sRNA21 and amir21 and **(B)** *Si*sRNA154 and amir154. Arabidopsis protoplasts were transformed with either pUC18-*At*MIR390a-RFP-*Si*sRNA or pUC18-*At*MIR390a-RFP-

amiRNA. Control protoplasts were treated with water. Total RNA was extracted 24 hptr and reverse transcribed into cDNA. The Arabidopsis housekeeping gene *Ubiquitin (UBC21, AT5G25760*) was used for normalization. Data show fold changes in the expression of *AT5G25350* and *AT4G32160* relative to the control sample as analyzed by quantitative Real-Time PCR. Bars represent the average of three independent biological replicates ± SD. The asterisks indicate a significant difference ($P < 0.05$) according to an unpaired Student's *t*-test.

Reference

Bargmann, B. O. R., & Birnbaum, K. D. (2009). Positive Fluorescent Selection Permits Precise, Rapid, and In-Depth Overexpression Analysis in Plant Protoplasts. *Plant Physiology*, *149*(3), 1231–1239. https://doi.org/10.1104/pp.108.133975

Carbonell, A., Takeda, A., Fahlgren, N., Johnson, S. C., Cuperus, J. T., & Carrington, J. C. (2014). New generation of artificial MicroRNA and synthetic trans-acting small interfering RNA vectors for efficient gene silencing in Arabidopsis. *Plant Physiology*, *165*(1), 15–29. https://doi.org/10.1104/pp.113.234989

Dai, X., Zhuang, Z., & Zhao, P. X. (2018). psRNATarget: A plant small RNA target analysis server (2017 release). *Nucleic Acids Research*, *46*(Web Server issue), W49–W54. https://doi.org/10.1093/nar/gky316

Šečić, E., Zanini, S., Wibberg, D., Jelonek, L., Busche, T., Kalinowski, J., Nasfi, S., Thielmann, J., Imani, J., Steinbrenner, J., & Kogel, K.-H. (2021). A novel plant-fungal association reveals fundamental sRNA and gene expression reprogramming at the onset of symbiosis. *BioMed Central Biology*, *19*(1), 171. https://doi.org/10.1186/s12915-021-01104-2
